# Core-block engineering enables control of ice recrystallisation inhibition in polymer nanoparticles

**DOI:** 10.1039/d6sc02659a

**Published:** 2026-05-18

**Authors:** Panagiotis G. Georgiou, Cigdem Buse Oral, Ho Fung Mack, Hubert Buksa, Chenrui Bao, Csilla György, Steven P. Armes, Matthew I. Gibson

**Affiliations:** a School of Mathematical and Physical Sciences, University of Sheffield Brook Hill Sheffield South Yorkshire S3 7HF UK panagiotis.georgiou@manchester.ac.uk s.p.armes@sheffield.ac.uk; b Department of Chemistry, University of Manchester Oxford Road Manchester Greater Manchester M13 9PL UK matt.gibson@manchester.ac.uk; c Manchester Institute of Biotechnology, University of Manchester 131 Princess Street Manchester Greater Manchester M1 7DN UK

## Abstract

Ice-binding proteins (IBPs) are potent ice recrystallisation inhibitors (IRIs) that have inspired the development of synthetic cryoprotectants, spanning small molecules to polymers, but there are only a few soft nanomaterial IRI's reported. Polymerisation-induced self-assembly (PISA) derived nanoparticles are an appealing biomaterial scaffold due to their tuneability and synthetic scalability. Here we demonstrate for the first time that the internal hydrophobic core of PISA-derived nanoparticles controls the observable IRI activity, rather than the expected solvent-exposed hydrophilic corona. We systematically map the chemical and compositional design space of spherical PISA nanoparticles to parameterise the key features for this activity. Switching from glassy to soft nanoparticle cores leads to significantly enhanced IRI activity, whereas crosslinking the same soft cores eliminated all activity. These results establish a new framework for precision-tuneable IRI-active colloids and unlock opportunities to control IRI through modulation of the internal structure of nanoparticles without adapting the surface.

## Abstract 2

We demonstrate that engineering the core blocks of nanoparticles derived from polymerization-induced self assembly can tune the ice-recrystallisation inhibition activity: a previously unexplored route to modulate this biomimetic function.

## Graphical abstract

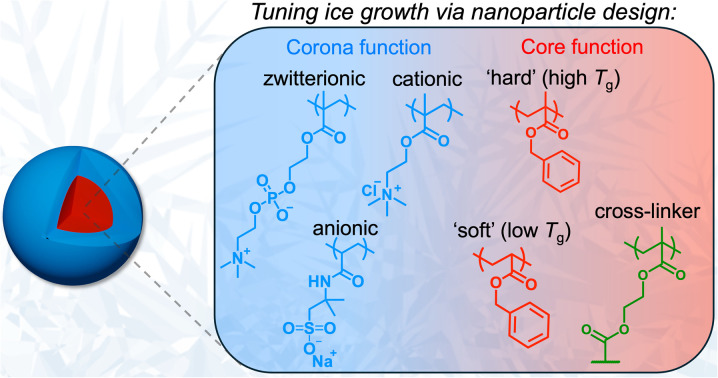

## Introduction

Nature has evolved macromolecules based on proteins,^1^ glycoproteins^2^ and lipo-polysaccharides^3^ that can modulate the formation and growth of ice, thus enabling life to survive even at sub-zero temperatures. Ice-binding proteins (IBPs) and antifreeze proteins (AFPs) have been extensively studied for biotechnological applications such as cryopreservation,^4–9^ food additives^10,11^ and anti-icing surfaces.^12,13^ However, such proteins are relatively expensive and have not yet found widespread commercial application. Synthetic analogues are now emerging that can replicate some, or all, of the properties of IBP. For example, poly(vinyl alcohol)^14,15^ can form hydrogen bonds with ice,^16^ amphiphilic molecules^17,18^ can disrupt the ice/water interface, and peptides and 2D materials have been discovered which mimic some aspects of IBP function.^19^ Ice recrystallisation inhibition (IRI) is the process of suppressing ice crystal growth (Ostwald ripening). This specific property has emerged as being amenable to being replicated by synthetic materials, in contrast to the other IBP properties of thermal hysteresis (freezing point depression) or dynamic ice shaping, which each require specific ice crystal face binding. Ice-binding mimics have been used for cryopreservation to mitigate either transient warming events or ice growth during thawing.^20^

Although synthetic materials typically exhibit lower IRI activity than IBPs, one traditional design strategy involves truncating or simplifying the ice-binding unit and displaying it in a multivalent fashion, as demonstrated with oligopeptides.^21–25^ More recently, attention has shifted toward constructing higher-order nanostructures.^26^ Early examples, including low grafting density nanoparticles based on polymers or peptides grafted onto gold nanoparticles,^27,28^ dendrimer-linked ice-binding proteins^29^ or coacervates,^30^ showed no discernible improvement in IRI activity compared to their hydrophilic coronal components. In contrast, Gibson and co-workers reported that diblock copolymer nanoparticles prepared *via* polymerisation-induced self-assembly (PISA) exhibited remarkable IRI activity upon nanoparticle formation, despite comprising water-contacting coronal blocks with no intrinsic IRI activity.^31^

Importantly, PISA is an established technology for the efficient synthesis of colloidally stable block copolymer nanoparticles with well-defined morphologies directly in aqueous media at relatively high solids.^32–38^ Moreover, free poly(ethylene glycol) (PEG) chains exhibited no discernible IRI even at 40 mg mL^−1^, whereas poly(ethylene glycol)-stabilised nanoparticles prepared *via* PISA completely inhibited ice growth at concentrations as low as 5 mg mL^−1^. Similar enhancements were observed for other IRI-inactive corona blocks. For example, poly(vinyl alcohol)-stabilised nanoparticles also displayed greater activity than the corresponding poly(vinyl alcohol) homopolymer alone.^39^ Guo and co-workers replicated these findings and emphasised the influence of the packing parameter on IRI behaviour.^40,41^ However, they did not investigate the role of either the coronal or core-forming blocks. These prior studies indicate that PISA enables the convenient generation of colloidal nanoparticles with emergent IRI activity but a physically reasonable explanation for such observations is currently lacking.

Herein, we examine the independent roles played by the coronal and core-forming blocks in determining ice recrystallisation inhibition (IRI) for PISA-derived spherical nanoparticles. We demonstrate that the coronal block exerts minimal influence over IRI activity, regardless of whether it has anionic, cationic, or zwitterionic character. Remarkably, we show for the first time that engineering the core block – normally considered to be a passive component since it is not in direct contact with either water or ice – can induce profound changes in IRI performance. This study highlights the role of the glass transition temperature in determining the potency of polymer-based cryoprotectants. We show that even a modest increase in core-block rigidity by crosslinking ‘turns off’ IRI. These findings suggest new design space for tuning IRI activity *via* nanoparticle core chemistry, regardless of the coronal block, and raise fundamental questions about how macromolecular assemblies modulate crystallisation processes.

## Results and discussion

**Scheme 1:**
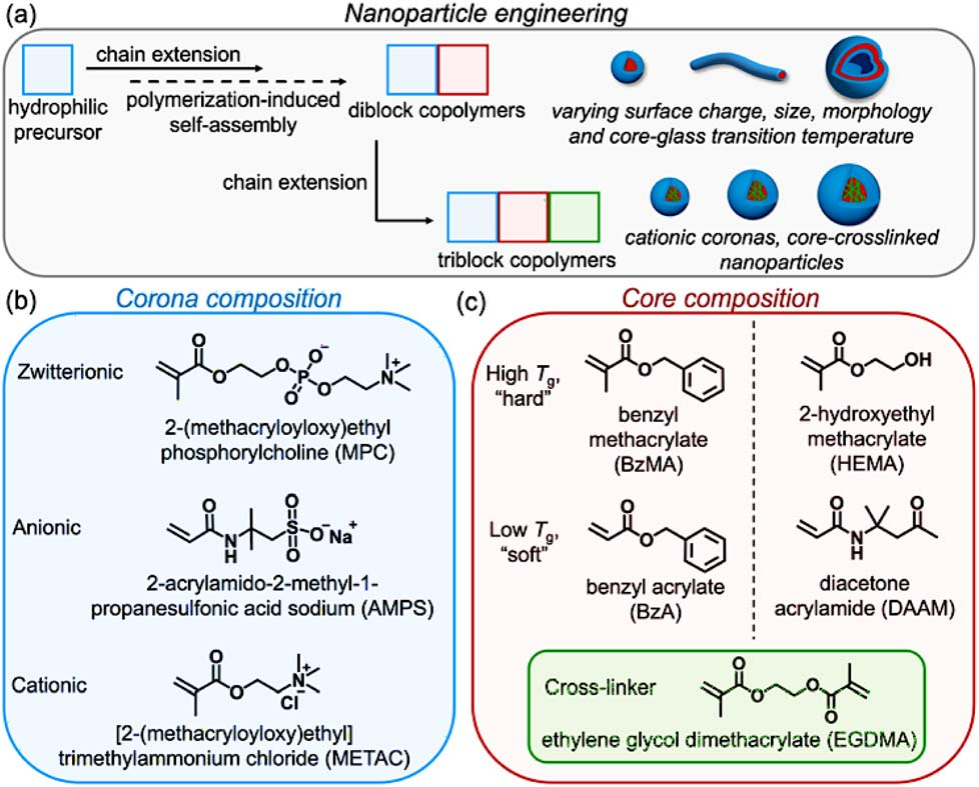
Chemical approach and design principles for ice recrystallisation inhibition-active copolymer nanomaterials prepared by aqueous polymerisation-induced self-assembly (PISA). A nanoparticle library is generated by (a) varying the nature of the coronal block (zwitterionic, anionic or cationic), (b) core-forming block composition (molar mass and glass transition temperature, *T*_g_), and (c) the copolymer morphology (spheres, worms or vesicles, depending on the target diblock compositions). In addition, cationic linear spherical nanoparticles based on PMETAC-PBzA can be core-crosslinked using ethylene glycol dimethacrylate (EGDMA) to yield PMETAC-PBzA-PEGDMA triblock copolymer nanoparticles.

A library of diblock copolymer nanoparticles was constructed to investigate how the coronal and core-forming blocks influence IRI performance (Scheme 1a). Rather than exploring worms or vesicles – whose influence on IRI has been explored previously^31,40^ – we chose to focus exclusively on spherical diblock copolymer nanoparticles. The following water-soluble corona-forming precursors were selected: poly(2-(methacryloyloxy)ethyl phosphorylcholine), PMPC (zwitterionic), poly(sodium 2-acrylamido-2-methyl-propanesulfonate), PAMPS (anionic) and poly[2-(methacryloyloxy)ethyl] trimethylammonium chloride, PMETAC (cationic) (Scheme 1b). We have previously demonstrated that zwitterionic coronas are highly effective for producing salt-tolerant diblock copolymer nanoparticles.^42,43^ Recently, György and co-workers reported that PMPC-stabilised diblock copolymer nanoparticles can be prepared *via* reversible addition–fragmentation chain transfer (RAFT) aqueous dispersion polymerisation of 2-hydroxyethyl methacrylate (HEMA) in the presence of salt, which reduces the aqueous solubility of both the HEMA monomer and the growing PHEMA chains.^44^ Salt was essential for the current study because ice recrystallisation inhibition (IRI) assays require salts within the buffer to generate a eutectic ice/water phase. In the absence of salt, false positives often occur and almost any polymer may appear to exhibit IRI activity.^45,46^

**Fig. 1:**
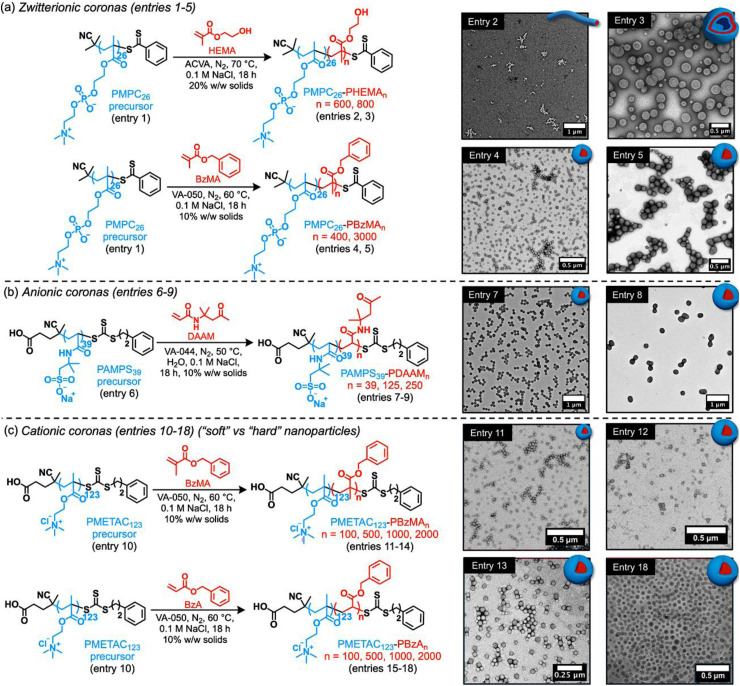
Aqueous PISA syntheses and TEM characterisation of zwitterionic, anionic or cationic diblock copolymer nanoparticles of variable size and morphology comprising either a hard or soft core-forming block. (a) Schematic representation of the preparation of PMPC_26_-PBzMA_*n*_ (*n* = 400 or 3000) spheres and PMPC_26_-PHEMA_*n*_ (*n* = 600 or 800) worms or vesicles. (b) Schematic representation of the preparation of PAMPS_39_-PDAAM_*n*_ (*n* = 39, 125, 250) spherical nanoparticles using an anionic PAMPS_39_ precursor. (c) Schematic representation of the preparation of hard (high *T*_g_) PMETAC_123_-PBzMA_*n*_ (100, 500, 1000 or 2000) and soft (low *T*_g_) PMETAC_123_-PBzA_*n*_ (100, 500, 1000 or 2000) spherical nanoparticles using a cationic hydrophilic precursor (PMETAC_123_). Abbreviations: PMPC = poly(2-(methacryloyloxy)ethyl phosphorylcholine), PAMPS = poly(sodium 2-acrylamido-2-methyl-propanesulfonate), PMETAC = poly[2-(methacryloyloxy)ethyl]trimethylammonium chloride, ACVA = 4,4′-azobis(4-cyanovaleric acid), VA-050 = 2,2′-azobis(2-methylpropionamidine)dihydrochloride, VA-044 = 2,2′-azobis[2-(2-imidazolin-2-yl)propane]dihydrochloride, BzMA = benzyl methacrylate, BzA = benzyl acrylate, HEMA = 2-hydroxyethyl methacrylate, DAAM = diacetone acrylamide [N. B. TEM images correspond to a selection of representative samples from each respective library].

Initially, a PMPC precursor was prepared by RAFT solution polymerisation using 2-cyano-2-propyl dithiobenzoate (PMPC_26_; *M*_n_ = 3.50 kDa, *M*_w_/*M*_n_ = 1.19). Details of this synthetic protocol are provided in the SI (see Experimental section, plus Fig. S1, S5 and entry 1 in Table 1). The RAFT aqueous dispersion polymerisation of HEMA was performed at 70 °C in the presence of 0.5 M NaCl while targeting a mean degree of polymerisation (DP) of either 600 or 800 at 20% w/w solids using 4,4′-azobis(4-cyanovaleric acid) (ACVA) initiator at a [PMPC_26_]/[ACVA] molar ratio of 5.0. High HEMA conversions (>98%) were confirmed by ^1^H NMR spectroscopy studies of aliquots taken from the reaction mixture (Fig. S2, entries 2 and 3 in Table 1). Size exclusion chromatography (SEC) analysis indicated unimodal molar mass distributions (Table 1 and Fig. S5). Copolymer dispersities remained relatively low (*M*_w_/*M*_n_ ≤ 1.50), which is consistent with previous reports for these diblock copolymers (Table 1).^44^ Dynamic light scattering (DLS) and transmission electron microscopy (TEM) studies suggested the formation of polydisperse worms and relatively uniform vesicles (Table 1, Fig. 1a and c) when targeting a PHEMA DP of 600 or 800, respectively.^32,47^ The same PMPC_26_ precursor was also chain-extended with benzyl methacrylate (BzMA) to yield PMPC-PBzMA diblock copolymer nanoparticles (entries 4 and 5). In this case, the RAFT aqueous emulsion polymerisation of BzMA was conducted at 60 °C in the presence of 0.1 M NaCl targeting a mean DP of either 400 or 3000 at 10% w/w solids. High BzMA conversions (>98%) were confirmed by ^1^H NMR spectroscopy studies of aliquots taken from the final reaction mixtures (Fig. S3, S4 entries 4 and 5 in Table 1). SEC analysis indicated unimodal molar mass distributions (Fig. 1a and entries 4 and 5 in Table 1 and Fig. S5). DLS and TEM analysis confirmed the formation of uniform spheres of either 50 or 150 nm diameter, respectively (Fig. 1 and entries 2 and 3 in Table 1 and Fig. S6). Aqueous electrophoresis studies confirmed *ζ*-potentials close to zero owing to the zwitterionic coronal chains (Table 1).

**Table 1:** Summary of the characterisation data obtained for all homopolymer precursors and diblock copolymers in this study

Entry number	Polymer composition	[Solids] (% w/w)	Total conv. (%)^a^	*M*_n,SEC_ (kDa)^b^	*M*_w_/*M*_n_^b^	*D*_h_ (nm)^c^	PD^c^	Morphology^d^	*ζ*-potential (mV)/pH 6.8^e^
1	PMPC_26_ precursor	40	82	3.50	1.19	n/a	n/a	Unimers	n/a
2	PMPC_26_-PHEMA_600_	20	>99	187.5	1.47	590	0.31	Worms	−3.1
3	PMPC_26_-PHEMA_800_	20	>99	304.7	1.43	262	0.16	Vesicles	+0.1
4	PMPC_26_-PBzMA_400_	10	98	84.8	1.25	46	0.09	Spheres	−2.1
5	PMPC_26_-PBzMA_3000_	10	>99	545.8	2.01	146	0.08	Spheres	−1.4
6	PAMPS_39_ precursor	50	78	7.50	1.35	n/a	n/a	Unimers	n/a
7	PAMPS_39_-PDAAM_39_	10	>99	64.0	1.40	92	0.02	Spheres	−46
8	PAMPS_39_-PDAAM_125_	10	>99	150.8	1.78	200	0.01	Spheres	−48
9	PAMPS_39_-PDAAM_250_	10	>99	446.7	1.78	300	0.01	Spheres	−45
10	PMETAC_123_ precursor	40	82	8.90	1.19	n/a	n/a	Unimers	n/a
11	PMETAC_123_-PBzMA_100_	10	>99	n/a*	n/a*	34	0.18	Spheres	+37
12	PMETAC_123_-PBzMA_500_	10	>99	n/a*	n/a*	57	0.17	Spheres	+30
13	PMETAC_123_-PBzMA_1000_	10	>99	n/a*	n/a*	89	0.06	Spheres	+31
14	PMETAC_123_-PBzMA_2000_	10	>99	n/a*	n/a*	117	0.10	Spheres	+31
15	PMETAC_123_-PBzA_100_	10	>99	n/a*	n/a*	67	0.25	Spheres	+35
16	PMETAC_123_-PBzA_500_	10	>99	n/a*	n/a*	79	0.17	Spheres	+36
17	PMETAC_123_-PBzA_1000_	10	>99	n/a*	n/a*	95	0.18	Spheres	+31
18	PMETAC_123_-PBzA_2000_	10	>99	n/a*	n/a*	151	0.19	Spheres	+31

a Determined by ^1^H NMR spectroscopy.

b Apparent number-average molar mass, *M*_n_, and dispersity (*M*_w_/*M*_n_) calculated by SEC [*PMETAC-based diblock copolymers were not amenable to SEC analysis owing to their insolubility in common SEC eluents].

c *D*_h_ is the *z*-average hydrodynamic diameter and PD is the polydispersity index, as reported by DLS.

d Morphologies assigned based on either conventional TEM (stained using 0.75% w/w uranyl formate) or cryo-TEM images.

e *ζ*-potentials were determined by aqueous electrophoresis at pH 6.8 [N. B. All copolymers are described in terms of their target diblock composition].

Next, we explored the synthesis of spherical nanoparticles using poly(sodium 2-acrylamido-2-methyl-propanesulfonate) (PAMPS) as an anionic coronal block. A PAMPS_39_ precursor (*M*_n_ = 7.50 kDa, *M*_w_/*M*_n_ = 1.35) was first prepared by RAFT aqueous solution polymerisation of AMPS using 4-cyano-4-(phenylcarbonothioylthio) pentanoic acid. Further details of this synthetic protocol are provided in the SI (see Experimental section, plus Fig. S7, S9 and entry 6 in Table 1). Subsequently, the RAFT aqueous dispersion polymerisation of diacetone acrylamide (DAAM) was conducted at 50 °C in the presence of 0.1 M NaCl targeting a mean DP of 39, 125 or 250 at 10% w/w solids (Fig. 1b). We have previously reported that such aqueous PISA syntheses can tolerate the presence of salt.^31,39,48^ High DAAM conversions (>99%) were confirmed in all cases *via*^1^H NMR spectroscopy studies (Fig. S8, entries 6–9 in Table 1). SEC analysis revealed unimodal molar mass distributions (Table 1 and Fig. S9) but copolymer dispersities were relatively high (*M*_w_/*M*_n_ ≥ 1.80) when targeting PDAAM DPs of 39, 125 or 250. DLS and TEM analysis confirmed the formation of well-defined spheres of 92, 200 or 300 nm diameter, respectively (Fig. 1b, S10 and Table 1). Aqueous electrophoresis analysis indicated strongly negative *ζ*-potential of approximately −45 mV owing to the anionic sulfonate groups on the PAMPS coronal block (Table 1).

Lastly, we prepared cationic nanoparticles with poly[2-(methacryloyloxy)ethyl]trimethylammonium chloride (PMETAC) as a coronal block using a synthetic protocol recently reported by Buksa and co-workers.^49^ In this case, PMETAC precursor was synthesised by RAFT solution polymerisation in methanol (PMETAC_123_, *M*_n_ = 8.9 kDa, *M*_w_/*M*_n_ = 1.10) and added salt was required to obtain well-defined colloidally stable spheres. Again, tuning the core-forming block DP conferred control over the mean particle diameter^49^ and further details are provided in the SI (see Experimental section, plus Fig. S11, S12 and entry 10 in Table 1). RAFT aqueous emulsion polymerisation of benzyl methacrylate (BzMA) was conducted at 60 °C in the presence of 0.1 M NaCl, targeting mean DPs of 100, 500, 1000 or 2000 at 10% w/w solids. ^1^H NMR analysis in DMSO-*d*_6_ showed high BzMA conversions (>99%) in all cases (Fig. S13, entries 11–14 in Table 1). DLS and TEM analysis confirmed the formation of uniform spherical nanoparticles with mean hydrodynamic diameters of approximately 34, 57, 89, and 117 nm for DPs of 100, 500, 1000 or 2000, respectively (Fig. S14 and entries 11–14 in Table 1).

Benzyl acrylate (BzA) was also employed as an alternative core-forming monomer to prepare PMETAC-PBzA spherical nanoparticles under identical conditions (60 °C, 0.1 M NaCl, 10% w/w solids) when targeting the same mean PBzA DPs of 100, 500, 1000 or 2000. High BzA conversions (>99%) were achieved in all cases (entries 15–18 in Table 1). DLS and cryo-TEM analysis confirmed the formation of uniform spheres ranging from 67 to 151 nm (Fig. S15 and entries 15–18 in Table 1). DLS analysis indicated higher polydispersities (PD ≥ 0.20) for such PMETAC-PBzA nanoparticles compared to the corresponding PMETAC-PBzMA nanoparticles. Aqueous electrophoresis studies confirmed the cationic character of both PMETAC-PBzMA and PMETAC-PBzA nanoparticles, with positive *ζ*-potentials of approximately +30 mV. This is consistent with the quaternary ammonium groups in the PMETAC coronal block (Table 1).

**Fig. 2:**
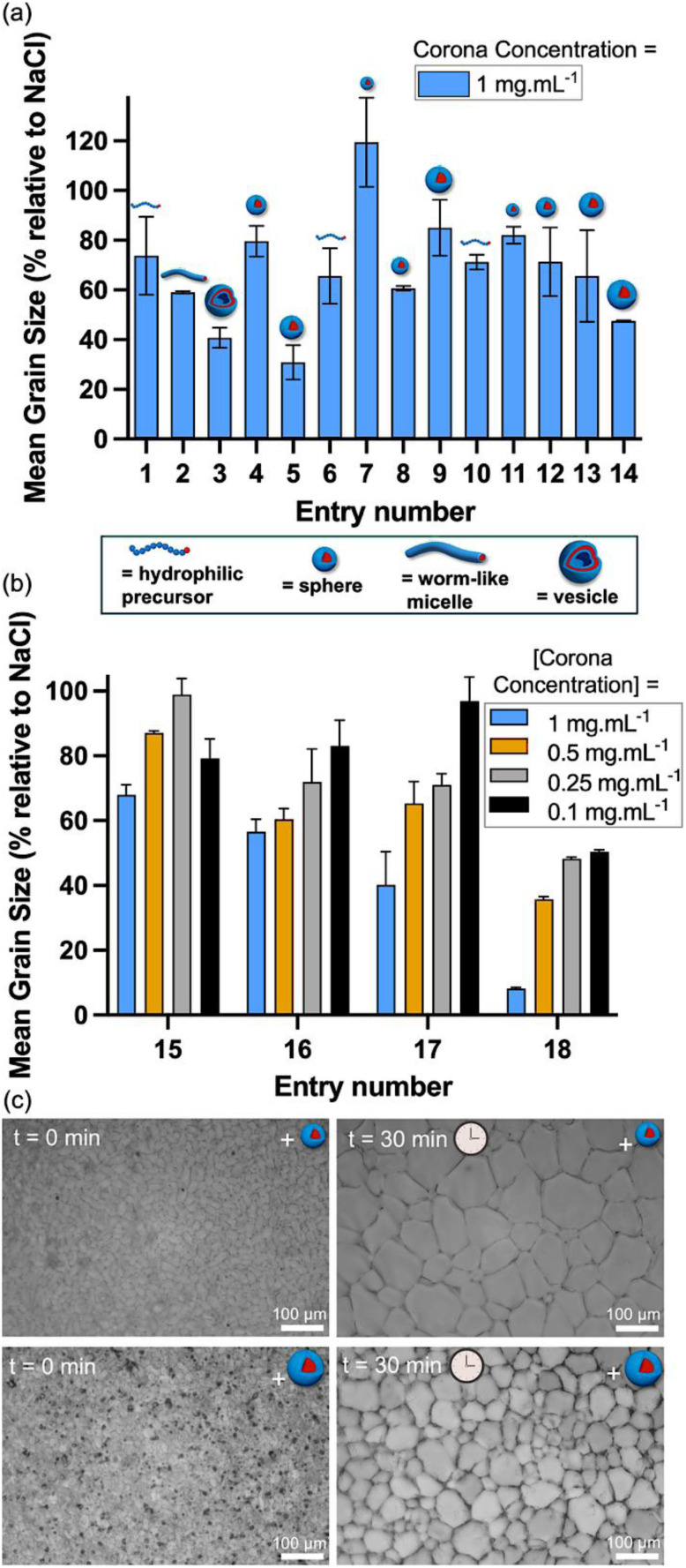
(a) Summary of ice recrystallisation inhibition (IRI) activity for zwitterionic (entries 1–5) block copolymer nanoparticles, anionic (entries 6–9) block copolymer nanoparticles and cationic diblock copolymer nanoparticles with high *T*_g_ PBzMA cores (entries 11–14) and low *T*_g_ PBzA cores (entry 18) at a coronal block concentration of 1.0 mg mL^−1^. (b) Summary of IRI activity for cationic PMETAC_123_-PBzA_*n*_ nanoparticles (*n* = 100, 500, 1000 or 2000) at a coronal PMETAC block concentration of 0.10, 0.25, 0.50 or 1.0 mg mL^−1^. Error bars are ±SD based on a minimum of three repeats. The percent mean grain size (MGS) is reported relative to that obtained for a 0.1 M NaCl control. (c) Representative optical micrographs recorded for the ‘splat’ assay after annealing for 30 min at −8 °C in the presence of 0.1 M NaCl for [PMETAC_123_] = 0.50 mg mL^−1^ for PMETAC_123_-PBzA_100_ (top) and PMETAC_123_-PBzA_2000_ (bottom).

With this library of nanoparticles to hand, the ‘splat’ assay was used to evaluate IRI activity.^45,50^ This involves forming a thin (<10 micron) wafer of ice, which is annealed at sub-zero temperatures to allow recrystallisation (ice growth). After 30 min each wafer is imaged, and the mean ice crystal size relative a control (0.1 M NaCl) solution is determined. Several samples are also analysed manually for quality control. To avoid bias when comparing nanoparticles of differing size, all data are reported in terms of the concentration of the coronal polymer chains; this approach enables IRI activity to be compared to that obtained when using the coronal chains alone (*i.e.*, in the absence of any nanoparticles). Fig. 2a shows the mean ice grain size observed for nanoparticle entries 1 to 14 at 1 mg mL^−1^ revealing potent IRI activity for many samples (see also Fig. S17 for IRI activities observed at 2.5 and 5.0 mg mL^−1^). In general, the smallest nanoparticles (<100 nm diameter) exhibited the weakest IRI activity. For larger nanoparticles (>200 nm diameter), consistently higher IRI activity (smaller MGS values) were observed, which is consistent with our earlier studies involving PISA-derived nanoparticles.^31,39^ A comparison of entries 4 and 5 (zwitterionic) with entries 12 and 14 (cationic) provides further insight, as these systems share identical. At comparable sizes, entries 4 and 12 exhibit similar IRI activity (MGS ∼70%). Interestingly, the larger entry 5 shows higher inhibition (MGS ∼30%) compared to entry 14 (MGS ∼50%), suggesting that while core chemistry is consistent, the zwitterionic corona may offer slight advantages in efficiency at higher degrees of polymerisation. An increase in IRI activity is also observed for nanoparticles with relatively high coronal densities, such as worms and vesicles (entries 2 and 3).^31,40^ In negative control experiments, all polymer precursors exhibited negligible IRI activity at 1.0 mg mL^−1^ (entries 1, 6, and 10), which is comparable to the low activity observed for the smallest nanoparticles in each series.

Comparison of nanoparticles comprising zwitterionic, anionic or cationic coronas indicated higher IRI activity compared to that for the coronal chains alone. However, there was no clear trend to suggest that a particular type of coronal block was more active, which indicates that electrostatic interactions are not crucial in this context. Importantly, the versatile nature of PISA enables the chemical nature of the core-forming block to be easily adjusted. This aspect had not been systematically studied prior to the current study – this is no doubt because the nanoparticle cores are not in direct contact with ice, so the core-forming block was considered to be a passive component. Nevertheless, we undertook to compare the IRI activity for hard (high *T*_g_) (entries 11–14) and soft (low *T*_g_) (entries 15–18) nanoparticle cores. Interestingly, PMETAC_123_-PBzA_2000_ nanoparticles with soft PBzA cores (entry 18; *T*_g_ = 9.1 °C) was much more active (MGS ∼10%) than PMETAC_123_-PBzMA_2000_ with hard PBzMA cores (entry 14; *T*_g_ = 61.5 °C; MGS ∼50%). According to the literature, *T*_g_ is a proxy for chain mobility:^51^ diblock copolymer nanoparticles comprising a low *T*_g_ core-forming block are typically much more dynamic than those with high *T*_g_ cores, which allows more rapid exchange of individual copolymer chains between nanoparticles.^52–55^ A comparison of smaller nanoparticles (entries 11 *vs.* 15, 12 *vs.* 16, and 13 *vs.* 17) reveals that PBzA-core particles achieve similar or enhanced IRI activity compared to PBzMA-core particles at equivalent corona concentrations (Fig. 2a and b).

Individual amphiphilic copolymer chains could interact with the ice-rich phase, leading to stronger IRI activity. In this context, Graham *et al.* reported that amphiphilic polymers with segregated hydrophilic and hydrophobic domains possess IRI activity,^17^ while synthetic amphipathic poly(proline)s exhibit comparable performance.^56–58^ Hence, a relatively low concentration of PMETAC_123_-PBzA_2000_ chains could potentially influence IRI activity. If this is correct, it would explain why soft nanoparticles exhibit higher IRI activity. This parameter is plotted as a function of copolymer concentration for PMETAC_123_-PBzMA_2000_ (Fig. S18) and PMETAC_123_-PBzA_2000_ (Fig. S19), respectively. Thus, even when accounting for the relatively high mass fraction of the core-forming block, the nanoparticles are significantly more active than the PMETAC coronal chains alone on a weight for weight basis. Despite the expectation that longer hydrophobic blocks should minimize unimer exchange,^59^ the highest molecular-weight core block (PMETAC_123_-PBzA_2000_) exhibited greater IRI activity than their lower molecular-weight analogues (PMETAC_123_-PBzA_100_). This suggests that additional factors beyond core mobility and unimer exchange, such as core block length, interfacial presentation, or aggregated morphologies contribute to the enhanced IRI performance as we observe a consistent size-dependent trend across both high- and low-*T*_g_ series.

These findings indicate that engineering the nanoparticle cores is a hitherto unexplored approach for the modulation of IRI activity. In principle, it offers new chemistries for the identification of novel cryoprotective nanoparticles. Accordingly, three additional PMETAC_123_-PBzA_*n*_ (*n* = 100, 500 or 1000) nanoparticles were evaluated for their dose–dependent IRI activity (entries 15–17). As the mean nanoparticle diameter (which correlates with the core-forming block DP)^60^ is increased from 67 to 151 nm, a gradual increase in IRI activity is observed (Fig. 2b). Fig. 2c shows representative optical micrographs obtained for the ‘splat’ assay when using PMETAC_123_-PBzA_100_ (left) and PMETAC_123_-PBzA_2000_ (right) after 30 min annealing at −8 °C for [PMETAC_123_] = 0.5 mg mL^−1^. This IRI activity is lower than that achieved when using ice-binding proteins but higher than many small molecule and polymers reported in the literature.^46^ The key finding here is that each component of the nanoparticles is individually inactive, which suggests that any water-soluble polymer corona can be engineered to exhibit IRI by selecting an appropriate low *T*_g_ core-forming block for the nanoparticle synthesis.

The colloidal stability of PMETAC_123_-PBzA_1000_ and PMETAC_123_-PBzA_2000_ (entries 17, 18) nanoparticles after multiple freeze–thaw cycles was assessed by DLS (re S20). This technique – which is sensitive to the onset of aggregation, indicated minimal change in hydrodynamic diameter and DLS polydispersity in each case. This suggests that these soft nanoparticles do not form films on the surface of the ice crystals, otherwise one would not expect to recover individual nanoparticles after multiple freeze/thaw cycles. The PMETAC_123_-PBzMA_2000_ (entry 14) and PMETAC_123_-PBzA_2000_ (entry 18) nanoparticles were evaluated for specific ice crystal face binding in a nanolitre osmometer (Fig. S21 and S22). However, there was no evidence for faceting nor any changes in morphology. This indicates that such nanoparticles do not interact with a specific crystallographic plane of ice. Ben *et al.* proposed that amphiphilic IRI-active molecules located at the interface of ordered/disordered water may prevent efficient mass transport.^61,62^ A similar scenario might also be relevant for nanoparticles.

**Fig. 3:**
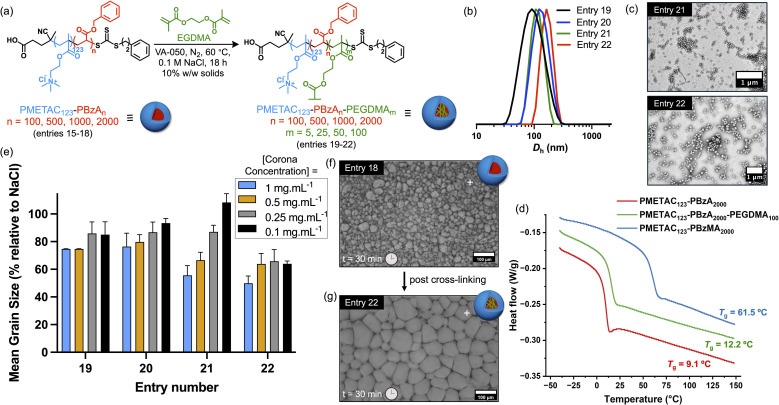
Core-crosslinking of PMETAC_123_-PBzA_*n*_ (*n* = 100, 500, 1000 or 2000) spherical nanoparticles and assessment of their ice recrystallisation inhibition (IRI) activity. (a) Synthesis of core-crosslinked PMETAC_123_-PBzA_100_-PEGDMA_5_ (entry 19), PMETAC_123_-PBzA_500_-PEGDMA_25_ (entry 20), PMETAC_123_-PBzA_1000_-PEGDMA_50_ (entry 21), PMETAC_123_-PBzA_2000_-PEGDMA_100_ (entry 22) triblock copolymer nanoparticles (see entries 19–22 in Table 2). (b) Intensity-weighted particle size distributions determined by DLS for PMETAC_123_-PBzA_*n*_-PEGDMA_*m*_ (*m* = 5, 25, 50 or 100) nanoparticles (entries 19–21). (c) Representative TEM images recorded for PMETAC_123_-PBzA_1000_-PEGDMA_50_ (entry 21) and PMETAC_123_-PBzA_2000_-PEGDMA_100_ (entry 22). (d) DSC curves recorded for PMETAC_123_-PBzA_2000_ (red trace), PMETAC_123_-PBzA_2000_-PEGDMA_100_ (green trace) and PMETAC_123_-PBzMA_2000_ (blue trace). (e) Summary of IRI activity for PMETAC_123_-PBzA_*n*_-PEGDMA_*m*_ (*n* = 100, 500, 1000 or 2000; *m* = 5, 25, 50 or 100) core-crosslinked nanoparticles at [PMETAC] of 0.10, 0.25 or 0.50 mg mL^−1^. Error bars are ±SD based on a minimum of three repeats. The percent mean grain size (MGS) was reported relative to a 0.1 M NaCl control. Representative optical micrographs from the ‘splat’ assay recorded after annealing for 30 min at −8 °C in 0.1 M NaCl at a fixed [PMETAC_123_] = 1.0 mg mL^−1^ for (f) PMETAC_123_-PBzA_100_ (top) and (g) PMETAC_123_-PBzA_2000_ (bottom).

To examine whether individual amphiphilic diblock copolymer chains influence the IRI activity, a series of soft PMETAC_123_-PBzA_*n*_ (*n* = 100, 500, 1000 or 2000) nanoparticles were core-crosslinked using ethylene glycol dimethacrylate (EGDMA) as a third block. Such syntheses involve RAFT aqueous emulsion polymerisation of EGDMA at 10% w/w solids using the corresponding linear PMETAC_123_-PBzA_*n*_ nanoparticles as seeds. To minimize the increase in *T*_g_ for the nanoparticle cores, the target PEGDMA DP was restricted to just 5% of the original PBzA DP. The resulting core-crosslinked nanoparticles (entries 19–22 in Table 2) were characterised by DLS and TEM (Fig. 3b and c, Table 2). Core-crosslinking prevented ^1^H NMR and SEC analysis but full EGDMA consumption was assumed after 18 h at 60 °C. DLS analysis indicated an increase in the mean nanoparticle diameter of ∼25 nm after crosslinking, while TEM analysis confirmed that the original spherical morphology was retained, as expected. Differential scanning calorimetry (DSC) was used to assess the *T*_g_ of the nanoparticle cores before and after crosslinking. This technique indicated only a modest increase in *T*_g_ of 3 °C for PMETAC_123_-PBzA_2000_-PEGDMA_100_ (Fig. 3d, S16 and Table 2), which suggests that the core-forming chains are only lightly crosslinked/branched. Nevertheless, crosslinking the nanoparticle cores removed all IRI activity in each case (Fig. 3e–g). This is consistent with our hypothesis that individual linear amphiphilic copolymer chains may be responsible for the observed IRI activity.^17,63^ However, we cannot rule out the possibility that nanoparticle deformability at the ice/water interface may also play a role. Clearly, further studies are warranted to identify the underlying mechanism(s), which should in turn enable optimisation of IRI activity.

**Table 2:** Summary of the characterisation data obtained for core-crosslinked triblock copolymer spherical nanoparticles prepared by RAFT aqueous emulsion polymerisation of EGDMA

Entry number	Copolymer composition	[Copolymer] (% w/w)	*D*_h_ (nm)^a^	PD^a^	*T*_g_ (°C)^b^
19	PMETAC_123_-PBzA_100_-PEGDMA_5_	10	90.6	0.23	21.3
20	PMETAC_123_-PBzA_500_-PEGDMA_25_	10	109.5	0.04	19.6
21	PMETAC_123_-PBzA_1000_-PEGDMA_50_	10	122.8	0.08	18.1
22	PMETAC_123_-PBzA_2000_-PEGDMA_100_	10	163.6	0.02	12.2

a *D*_h_ is the *z*-average hydrodynamic diameter and PD is the polydispersity index, as reported by dynamic light scattering.

b Determined by differential scanning calorimetry.

## Conclusions

We demonstrate for the first time that the nature of the core-forming block within diblock copolymer nanoparticles can influence their ice recrystallisation inhibition (IRI) activity. Construction of a library of nanoparticles comprising anionic, neutral or cationic coronas and either hard (high *T*_g_) or soft (low *T*_g_) core-forming blocks enabled us to elucidate the synthesis-structure–function relationship. The chemical nature of the coronal block alone had minimal impact on IRI, with all nanoparticles exhibiting greater IRI than the corresponding individual coronal chains. Importantly, nanoparticles with soft cores were significantly more IRI active than those with hard cores. Film formation on ice crystals was ruled out as a possible explanation, since freeze–thaw experiments suggested that freezing led to minimal nanoparticle deformation. Control experiments involving core-crosslinking of low *T*_g_ nanoparticles led to loss of IRI activity. This suggests that individual amphiphilic diblock copolymer chains might be important for IRI activity, but would require an unusual mechanism where unimer exchange is enhanced during the freezing. Further studies are required to elucidate the precise mechanism of IRI activity. Nevertheless, our observations indicate that the IRI activity of nanoparticles can be tuned by judicious design of the hydrophobic core-forming block, as opposed to the corona block. These results establish a framework for precision-tuneable colloids using scalable and cost-effective polymer materials by modulation of particle internal structure.

## Author contributions

P. G. G., C. B. O., H. F. M., H. B., C. B. and C. G. conducted experiments and analysed the data. P. G. G., S. P. A. and M. I. G. conceived the study and devised experiments. S. P. A., M. I. G. and P. G. G. directed the research. S. P. A. supervised P. G. G. P. G. G., M. I. G. and S. P. A. contributed to writing the manuscript.

## Conflicts of interest

There are no conflicts to declare.

## Supplementary Material

SC-OLF-D6SC02659A-s001

## Data Availability

All experimental data and procedures is within the supplementary information (SI). Supplementary information is available. See DOI: https://doi.org/10.1039/d6sc02659a.
