# Supplementary material for: Core-block engineering enables control of ice recrystallisation inhibition in polymer nanoparticles
Source: Chem Sci. 2026 May 18;17(25):12532–40. doi: 10.1039/d6sc02659a (PMC13182838; doi:10.1039/d6sc02659a)
Supplement: SC-017-D6SC02659A-s001 [file SC-017-D6SC02659A-s001.pdf]

Supplementary Information (SI) for Chemical Science.
This journal is © The Royal Society of Chemistry 2026

## Supplementary Information for:

# Core-block engineering enables control of ice recrystallisation inhibition in polymer nanoparticles

*Panagiotis G. Georgiou,*[*a] Cigdem Buse Oral,[b,c] Ho Fung Mack,[b,c] Hubert Buksa,[a] Chenrui Bao,[a] Csilla György,[a] Steven P. Armes,[*a] and Matthew I. Gibson[*b,c]*

[a] School of Mathematical and Physical Sciences, University of Sheffield, Sheffield, South Yorkshire, S3 7HF, UK.

[b] Department of Chemistry, University of Manchester, Oxford Road, Manchester, M13 9PL, UK.

[c] Manchester Institute of Biotechnology, University of Manchester, 131 Princess Street, Manchester, M1 7DN, UK.

*Corresponding Authors: s.p.armes@sheffield.ac.uk, panagiotis.georgiou@manchester.ac.uk and matt.gibson@manchester.ac.uk

**Table of Contents**

Materials and Methods....................S1
Materials....................S1
Characterisation Techniques....................S1
Experimental Procedures....................S3
Synthetic Procedures....................S5
Supporting Data for Supporting Ice Recrystallisation Inhibition/ Ice Shaping Data....................S18
References....................S22

## Materials and Methods

### Materials

Solvents (ACS grade) such as DMF, dioxane, acetone and methanol were purchased from Fischer Scientific and used without further purification. All chemicals were used as received. 2-Methacryloyloxyethyl phosphorylcholine (MPC, 97%), benzyl methacrylate (BzMA; 96%), benzyl acrylate (≥99.8%, BzA), 2-acrylamido-2-methyl-1-propanesulfonic acid sodium salt (AMPS, 50% w/w aqueous solution), diacetone acrylamide (DAAM, 99%) and [2-(methacryloyloxy)ethyl]- trimethylammonium chloride (METAC; 80% w/w aqueous solution) were purchased from Sigma Aldrich. Monomer of 2-hydroxyethyl methacrylate (HEMA; ≥99.5% triply distilled grade) monomer was kindly provided by GEO Specialty Chemicals (Hythe, UK) and was used as received. 2-Cyano-2-propyl benzodithioate (>97% HPLC), 4,4′-azobis(4-cyanopentanoic acid) (ACVA; >98%), 2,2′-azobis(2-methylpropionitrile) (AIBN, 98%), 2,2'-azobis(2-methylpropionamidine) dihydrochloride (VA-050, 97%) and sodium chloride (NaCl, ≥99.0%) were also purchased from Sigma Aldrich. 4-Cyano-4-(phenylcarbonothioylthio)pentanoic acid was synthesised in-house according to a literature protocol.[1] Formvar-carbon coated copper TEM grids were purchased from EM Resolutions. Methanol-$d_4$ ($CD_3OD$, 99.8%) and DMSO-$d_6$ ($(CD_3)_2SO$, 99%) were purchased from Goss Scientific Instruments Ltd, UK. Dialysis membranes (MWCO = 3.5 kDa) were purchased from Spectra/Por. Deionised water (pH 6.8) was obtained using an Elga Elgastat Oprion 3A water purification system with a 15.0 MΩ cm.

### Characterisation techniques

*NMR Spectroscopy.* $^1$H NMR and spectra were recorded in methanol-$d_4$, DMSO-$d_6$ or DMF-$d_7$ at 400 MHz using a Bruker Ascend™ 400 spectrometer. Proton chemical shifts are reported as $\delta$ in parts per million (ppm) and are expressed relative to tetramethylsilane (TMS) at $\delta = 0$ ppm.

*Size Exclusion Chromatography (SEC).* Size exclusion chromatography (SEC) analysis for $PAMPS_{39}$-$PDAAM_n$ (n = 39, 125, 250) diblock copolymers was performed using an Agilent 1260 Infinity GPC system equipped with an Agilent guard column (PLgel 5 μm) and two Agilent Mixed-C columns (PLgel 5 μm). Detection involved either a differential refractive index (RI) detector or a UV-visible detector (set to $\lambda = 305$ nm). The mobile phase was DMF (HPLC-grade) containing 10 mM LiBr at 60 °C at a flow rate of 1.0 mL min$^{-1}$. Number-average

molar mass ($M_n$), weight-average molar mass ($M_w$) and dispersities ($Đ = M_w/M_n$) were calculated using a series of near-monodisperse poly(methyl methacrylate) (PMMA) calibration standards ranging from 800 to 2,200,000 Da.

SEC analysis for $PMPC_{26}$-PHEMA and $PMPC_{26}$-PBzMA diblock copolymers was conducted at 35 °C using a 3:1 v/v chloroform/methanol eluent containing 2 mM LiBr at a flow rate of 1.0 mL.min$^{-1}$. The instrument setup comprised an Agilent 1260 GPC system, two Agilent PL gel 5 mm Mixed-C columns connected in series with a guard column, and a refractive index detector. Calibration was achieved using a series of ten near-monodisperse poly(methyl methacrylate) (PMMA) standards ranging from 2380 to 988 000 g.mol$^{-1}$.

Aqueous GPC analysis of the $PMPC_{26}$ precursor was conducted at 30 °C using an aqueous eluent containing 0.10 M $NaNO_3$, 0.02 M TEA, 0.05 M $NaHCO_3$, and 0.005 M $NaN_3$ (pH 8) at a flow rate of 1.0 mL.min$^{-1}$. The instrument setup comprised an Agilent 1260 GPC system; three PL Aquagel Mixed-H, OH-30, and OH-40 columns connected in series with a guard column; and a refractive index detector. Calibration was achieved using a series of ten near-monodisperse poly(ethylene glycol) (PEG) standards ranging from 240 to 912 800 g.mol$^{-1}$.

[N.b. All aqueous diblock copolymer dispersions of $PAMPS_{39}$-PDAAM, $PMPC_{26}$-PHEMA and $PMPC_{26}$-PBzMA were dialysed for 48 h to remove salt and lyophilised overnight prior to SEC analysis].

*Dynamic Light Scattering (DLS).* Measurements were performed using a Malvern Zetasizer Nano ZS instrument equipped with a 4 mW He–Ne 633 nm laser and an avalanche photodiode detector. Back-scattered light was detected at an angle of 173° and data were recorded at a copolymer concentration of 1% w/w at 25 °C. Malvern Zetasizer Software v7.11 was used to calculate z-average hydrodynamic diameters ($D_h$) *via* the Stokes-Einstein equation, which assumes perfectly monodisperse, non-interacting spherical particles. Data were averaged over at least three consecutive runs with at least ten measurements being recorded for each run.

*Transmission Electron Microscopy (TEM).* Aqueous dispersions of diblock or triblock copolymer nanoparticles were diluted to 0.10%. Copper-palladium TEM grids were surface-coated with a thin carbon film before being plasma glow-discharged for 30 seconds to produce a hydrophilic surface. An 11 μL droplet of a dilute aqueous dispersion of PMPC, PAMPS and PMETAC nanoparticles was deposited onto the surface of each TEM grid for 3 minutes before blotting with filter paper to remove excess liquid. An 11 μL droplet of a 0.75% w/v aqueous uranyl formate solution was then applied as a negative stain for 25 s prior to careful blotting

and drying using a vacuum hose. Imaging was performed at 80 kV using a FEI Tecnai G2 spirit instrument equipped with a Gatan 1k CCD camera.

*Cryogenic Transmission Electron Microscopy (Cryo-TEM).* Cryo-TEM study for $PMETAC_{123}$-$PBzA_{2000}$ was performed using an FEI Tecnai Arctica microscope operating at an accelerating voltage of 200 kV. Cryo-TEM samples were prepared by depositing 11 μL of a 1.0% w/w aqueous copolymer dispersion onto a plasma-treated Quantifoil® holey carbon-coated copper grid, followed by blotting for approximately 4 s and then plunging into liquid ethane to vitrify the sample using a Leica EM GP automatic plunge freezer (25 °C, 99% humidity). Transfer of the vitrified grids into a pre-cooled cryo-TEM holder was performed at −196 °C prior to analysis.

*Aqueous Electrophoresis.* To prepare copolymer dispersions for analysis, all nanoparticles were diluted to 0.01% w/w. A Malvern Instruments Zetasizer Nano ZS instrument was used for electrophoretic characterisation and mobilities were determined at 20 °C. All reported measurements were the average of at least five runs. Zeta potential was calculated from the corresponding electrophoretic mobilities ($\mu_E$) by using the Henry's correction of the Smoluchowski equation ($\mu_E = 4\pi\ \varepsilon_0\ \varepsilon_r\ \zeta\ (1+\kappa r)/6\pi\ \mu$)

*Differential scanning calorimetry (DSC).* Measurements were performed using a TA DSC25 Discovery series instrument operating from 0 to 150 °C at a heating/cooling rate of 10 °C.min$^{-1}$ using aluminum Tzero pans and standard lids. Instrument calibration was performed using an indium standard. All DSC analyses involved three heating/cooling cycles. [N.b. All aqueous block copolymer dispersions of $PMETAC_{123}$-$PBzA_{n}$ (n = 100, 2000), $PMETAC_{123}$-$PBzA_{100}$-$PEGDMA_{5}$, $PMETAC_{123}$-$PBzA_{500}$-$PEGDMA_{25}$, $PMETAC_{123}$-$PBzA_{1000}$-$PEGDMA_{50}$, $PMETAC_{123}$-$PBzA_{2000}$-$PEGDMA_{100}$. [N.b. All block copolymer dispersions were dialysed for 48 h to remove salt and lyophilised overnight prior to analysis].

**Experimental Procedures**

*Splat Ice Recrystallisation Inhibition Assay*. Splat cooling assays were performed as previously described by Tomczak *et al.*[2] Briefly, a 10 μL sample was dropped 1.40 m onto a chilled glass coverslip, resting on a thin aluminium block cooled to -78 °C placed on dry ice. Upon hitting the coverslip, a wafer with diameter of approximately 10 mm and thickness 10 μm was formed instantaneously. The glass coverslip was transferred onto the Linkam cryostage and held at -8 °C using liquid nitrogen for 30 minutes. Photographs were obtained using an Olympus CX 41

microscope with a UIS-2 20x/0.45/∞/0-2/FN22 lens and crossed polarisers (Olympus Ltd), equipped with a Canon DSLR 500D digital camera. Images were taken of the initial wafer (to ensure that a polycrystalline sample had been obtained) and again after 30 minutes. Images were analysed in Python using the Segment Anything Model (SAM) to identify ice grain boundaries and segment individual grains.[3] Grain areas were measured from each image, and the mean grain size (MGS) was determined. The average value and error were compared to that of [NaCl] = 0.1 M solution, as appropriate, as a negative control. The average value and error were compared to that of [NaCl] = 0.1 M solution, as appropriate, as a negative control.

*Ice Shaping using Nanoliter Osmometer.* An Otago nanoliter osmometer (Otago Osmometers, Dunedin, New Zealand) was used to measure polymer ice shaping. Briefly, 20 nL droplets of [PMETAC] = 0.1 mg/mL samples were suspended in type B immersion oil (Type B, cargille immersion oil) on a 6-well cooling plate using a microcapillary system.[4,5] The samples were rapidly frozen by cooling the osmometer to ~ −40 °C. A rapid temperature increase was then conducted until the melting point was reached, and then the temperature was gradually increased (0.01 °C.min$^{-1}$) until only one ice crystal remained. Just before the ice crystal melted, the temperature was decreased until a discernible growth of the ice crystal was observed. It was observed and photographed with an Olympus CX41 microscope equipped with a UIS 20x/0.45/*/0-2/FN22 lens (Olympus Ltd.) and a Canon EOS 1200D digital SLR camera.

*Colloidal stability studies during freeze-thaw cycles by DLS*. The colloidal stability for $PMETAC_{123}$-$PBzA_{1000}$ and $PMETAC_{123}$-$PBzA_{2000}$ was assessed following typical protocol.[6] Nanoparticle solutions were diluted in saline solution (0.1 M NaCl) at a final concentration of 1% w/w solids content (to final volume solution of 2 mL). Then, nanoparticle solution was submerged in liquid nitrogen for 10 min and left thaw at room temperature for 1 hour. 50 μL of nanoparticle solution was then taken and $D_h$ and PD changes were monitored by DLS. Five freeze-thaw cycles were conducted in total for each nanoparticle formulation.

**Synthetic Procedures**

*Polymerisation of 2-(methacryloyloxy)ethyl phosphorylcholine) using 2-cyano-2-propyl benzodithioate.* This synthetic protocol was previously reported by Beattie *et al.*[7] MPC monomer (35.0 g, 0.11 mol, 28 eq), 2-cyano-2-propyl benzodithioate (CPDB) (937.0 mg, 4.23 mmol, 1 eq), and AIBN initiator (139.0 mg, 0.85 mmol, 0.25 eq, CPDB/AIBN molar ratio = 5.0) were dissolved in ethanol (54.11 g) to afford a 40% w/w solution in a sealed round-bottom flask containing a magnetic stir bar. This flask was deoxygenated with a stream of $N_2$ gas for 30 min. The flask was heated to 70 °C with magnetic stirring for 140 min, and then the MPC polymerisation was quenched by exposing the reaction mixture to air while cooling the flask to 20 °C. A final MPC conversion of 82% was determined by comparing the integrated vinyl proton signal at 5.65−6.20 ppm with the oxymethylene signals assigned to the polymerised MPC units at 4.0−4.4 ppm using $^1$H NMR spectroscopy. The crude PMPC was precipitated twice into a 10-fold excess of a 17:1 v/v acetone/methanol mixture. Then the purified precursor was redissolved in deionised water and lyophilised overnight to produce a pink solid. The mean degree of polymerisation (and hence $M_{n,\ NMR}$) was calculated by end-group analysis by comparing the integrated dithiobenzoate end group (m, 7.25−7.50 ppm) with the two azamethylene protons assigned to the polymerised MPC units at 3.75 ppm assigned to the polymer side chain. $M_{n,\ NMR}$ = 7,900 Da ($DP_{PMPC}$ = 26). SEC analysis (3:1 chloroform/methanol) indicated an $M_{n,}$ of 3,600 Da and a $M_w/M_n$ of 1.32.

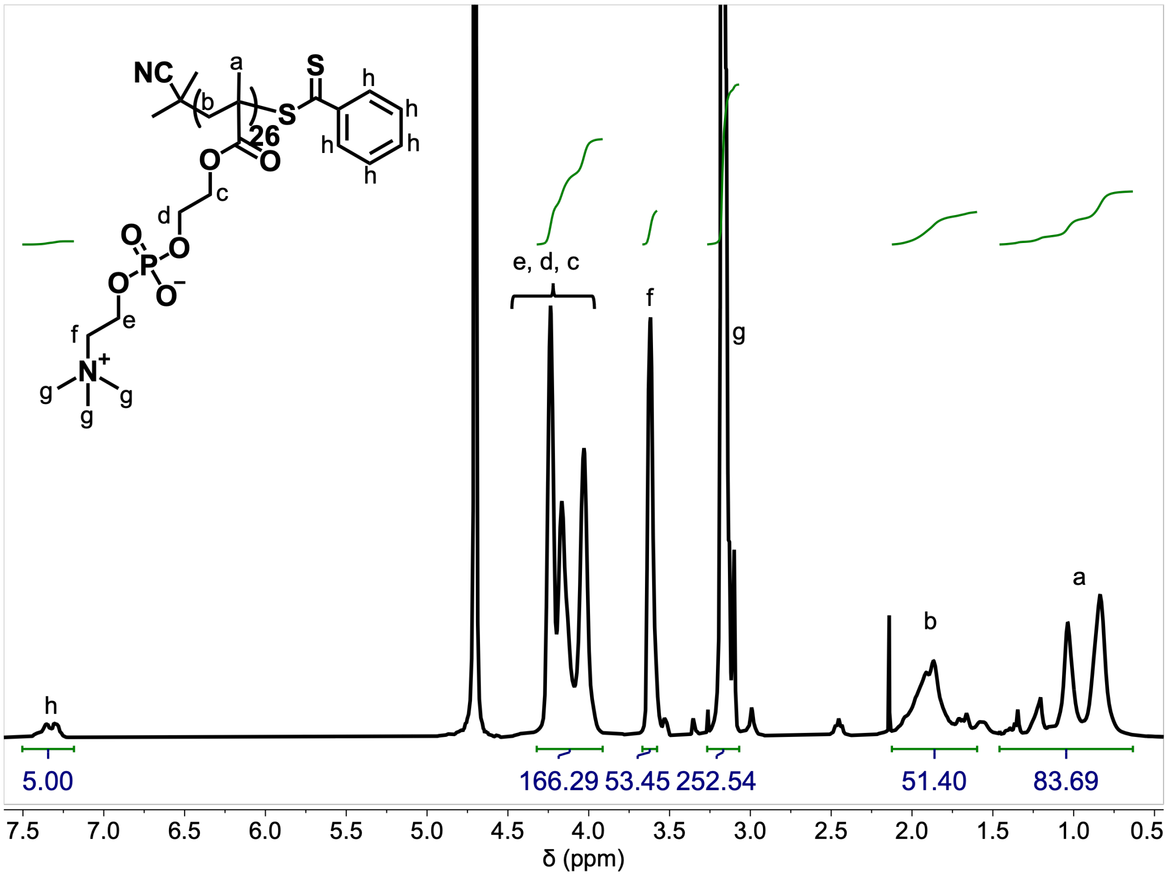


**Figure S1.** $^{1}H$ NMR spectrum ($D_2O$, 400 MHz) recorded for the $PMPC_{26}$ precursor.

*Synthesis of $PMPC_{26}$-$PHEMA_n$ nanoparticles by RAFT aqueous dispersion polymerisation in presence of 0.5 M NaCl.* A typical synthetic protocol for the preparation of *$PMPC_{26}$-$PHEMA_{600}$* nanoparticles at 20% w/w solids by RAFT aqueous dispersion polymerisation in 0.5 M NaCl is as follows. To a 6 mL glass vial containing a stirring bar, $PMPC_{26}$ precursor (100 mg, 0.0125 × $10^{-3}$ mmol, 1 eq), HEMA (976 mg, 7.50 mmol, 600 eq) and ACVA (0.9 mg, 0.003 × $10^{-3}$ mmol, 0.25 eq) were added and dissolved in 0.5 M NaCl (4.31 mL). The vial was sealed with a rubber septum and the reaction solution was purged with $N_2$ gas for 20 min. The vial was then placed into an aluminum heating block that had been pre-heated to 70 °C. After 18 h, the polymerisation was quenched by immersing the glass vial in liquid nitrogen and exposing its contents to air. An aliquot was removed for $^{1}H$ NMR spectroscopy studies in $CD_3OD$ and SEC analysis in 3:1 chloroform/methanol. TEM and DLS analyses were performed on samples after dilution to an appropriate copolymer concentration. This synthetic protocol was repeated for [HEMA]/[$PMPC_{26}$] molar ratio of 800. Nanoparticle samples were further diluted with deionised water to reach 0.1 M NaCl prior to splat ice recrystallisation inhibition assay analysis. In each case, relatively high monomer conversions were confirmed by $^{1}H$ NMR analysis, as indicated by the disappearance of the HEMA vinyl signals at 5.65−6.20 ppm.

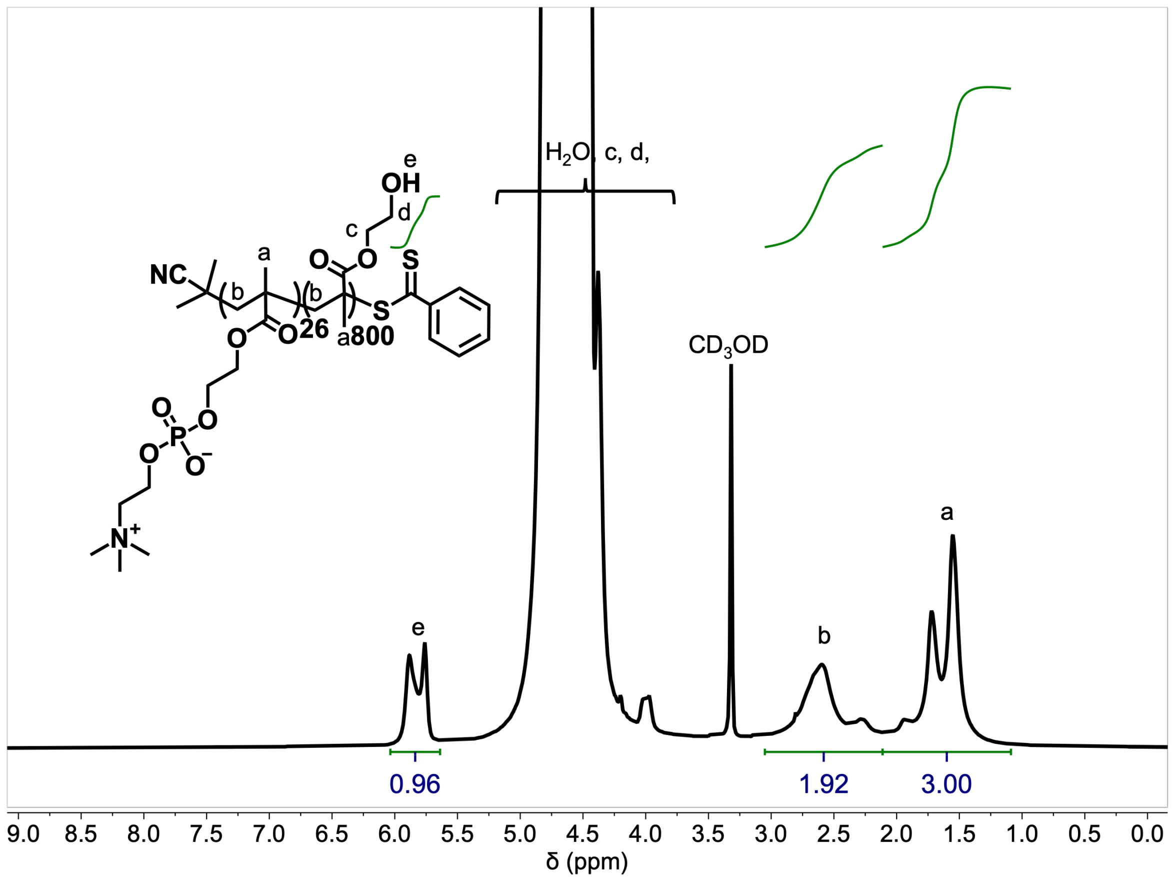


**Figure S2.** $^{1}H$ NMR spectrum ($CD_3OD$, 400 MHz) recorded for the $PMPC_{26}$-$PHEMA_{800}$.

*Synthesis of $PMPC_{26}$-$PBzMA_n$ nanoparticles by RAFT aqueous emulsion polymerisation in presence of 0.1 M NaCl.* A typical synthetic protocol for the preparation of *$PMPC_{26}$-$PBzMA_{400}$* nanoparticles at 10% w/w solids by RAFT aqueous dispersion polymerisation in 0.1 M NaCl is as follows. To a 6 mL glass vial containing a stirring bar, $PMPC_{26}$ precursor (50 mg, 0.0625 × $10^{-3}$ mmol, 1 eq), BzMA (441 mg, 2.50 mmol, 400 eq) and VA-050 (0.4 mg, 0.002 × $10^{-3}$ mmol, 0.25 eq) were added and dissolved in 0.1 M NaCl (4.42 mL). The vial was sealed with a rubber septum and the reaction solution was purged with $N_2$ gas for 20 min. The vial was then placed into an aluminum heating block that had been pre-heated to 60 °C. After 18 h, the polymerisation was quenched by immersing the glass vial in liquid nitrogen and exposing its contents to air. An aliquot was removed for $^{1}H$ NMR spectroscopy studies in $CD_3OD$ and SEC analysis in 3:1 chloroform/methanol. TEM, DLS and ζ-potential analyses were performed on samples after dilution to an appropriate copolymer concentration. This synthetic protocol was repeated for [BzMA]/[$PMPC_{26}$] molar ratio of 3000. In each case, relatively high monomer conversions were confirmed by $^{1}H$ NMR analysis, as indicated by the disappearance of the BzMA vinyl signals at 5.65−6.20 ppm.

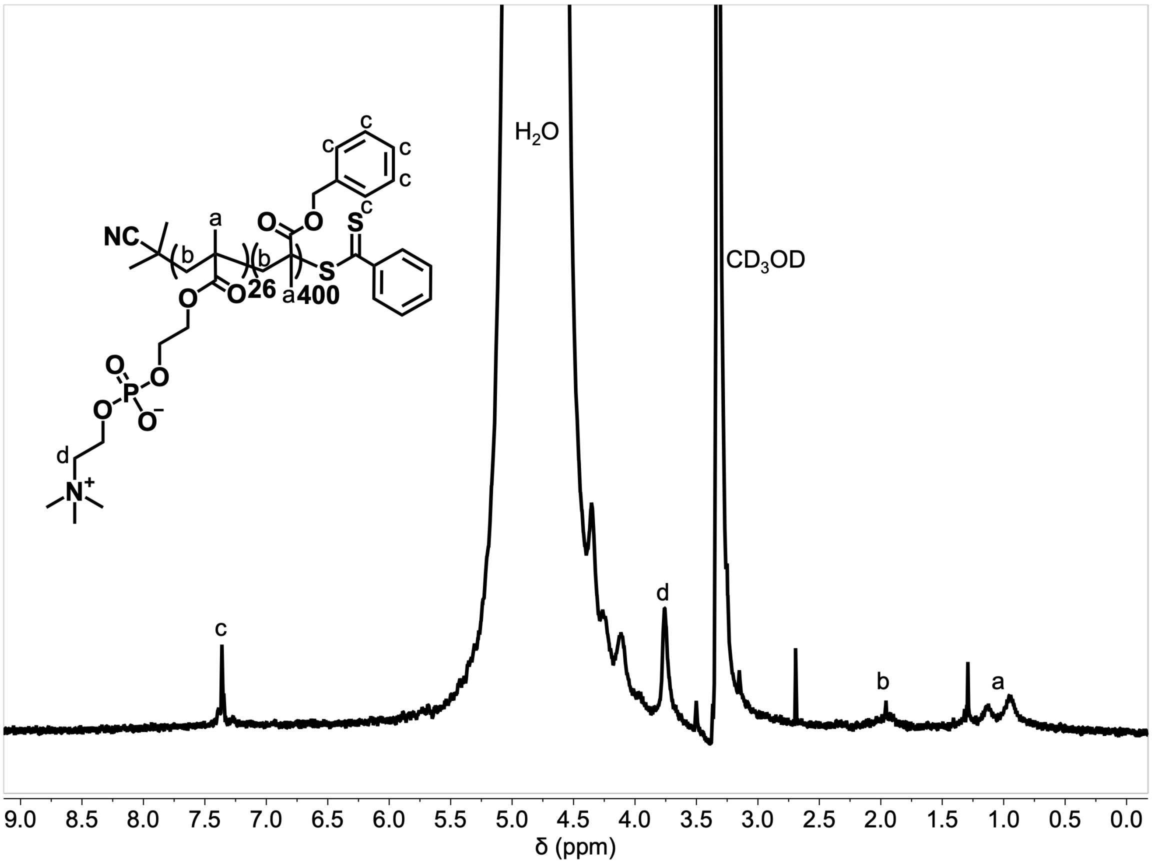


**Figure S3.** $^1$H NMR spectrum ($CD_3OD$, 400 MHz) recorded for the PMPC$_{26}$-PBzMA$_{400}$.

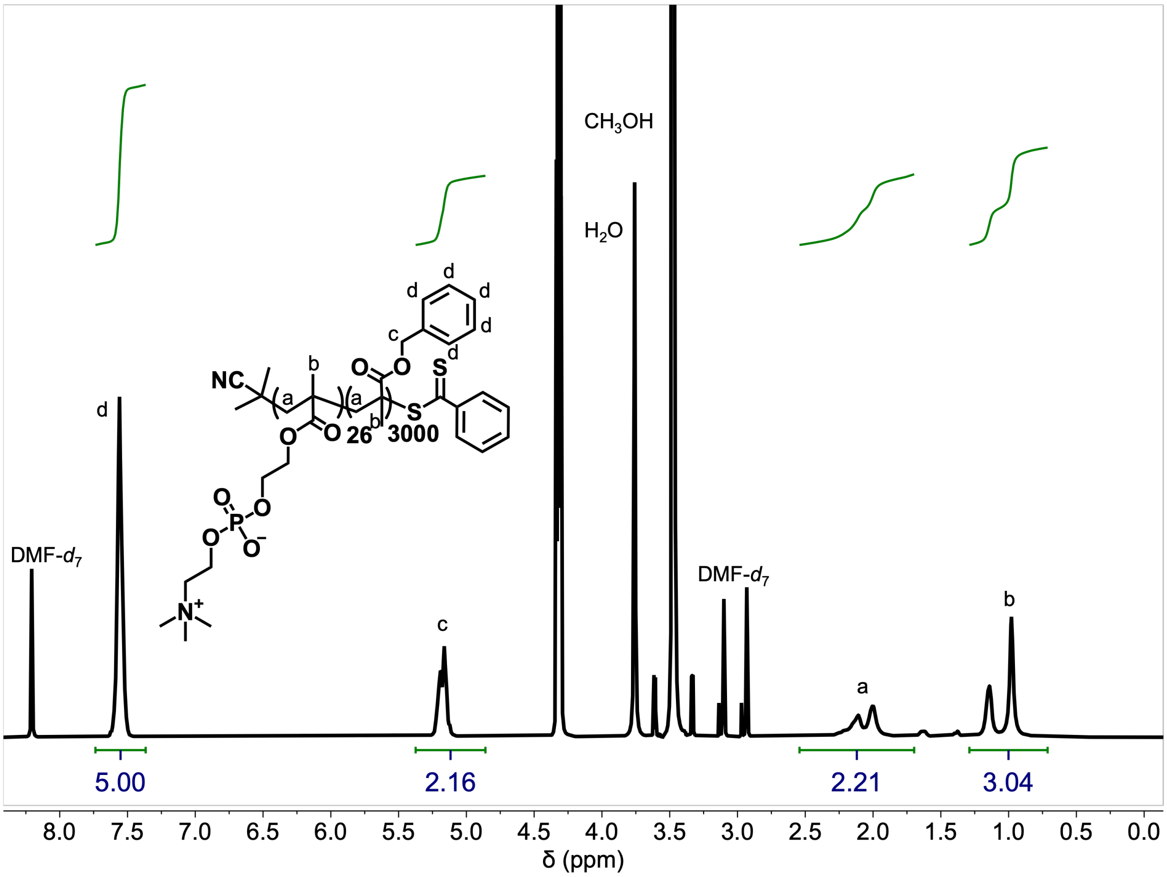


**Figure S4.** $^1$H NMR spectrum (DMF-$d_7$, 400 MHz) recorded for the PMPC$_{26}$-PBzMA$_{400}$.

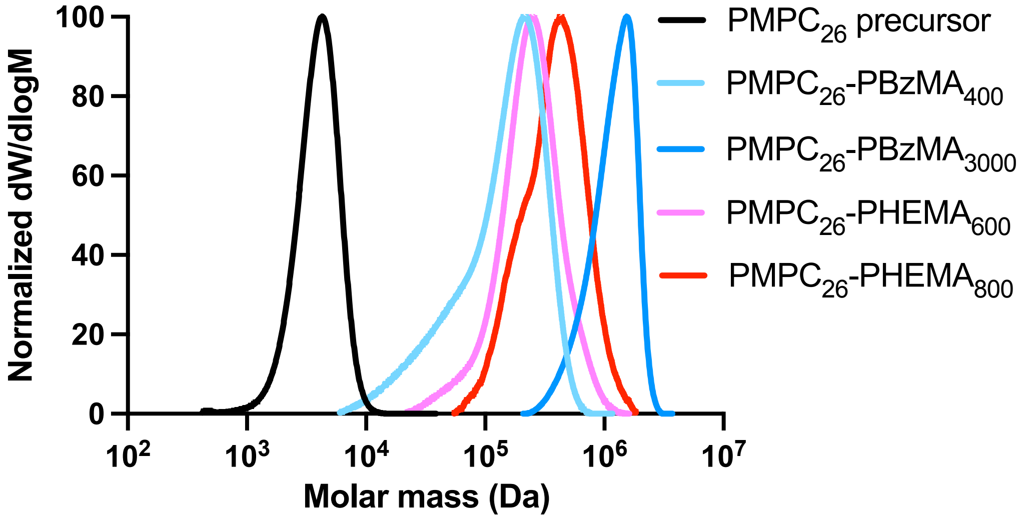


**Figure S5.** Normalised SEC curves (refractive index detector) recorded for a series of PMPC$_{26}$-PHEMA$_n$ (n = 600, 800), PMPC$_{26}$-PBzMA$_n$ (n = 400, 3000) diblock copolymers and the corresponding PMPC$_{26}$ homopolymer precursor (black trace) using 3:1 chloroform/methanol as eluent.

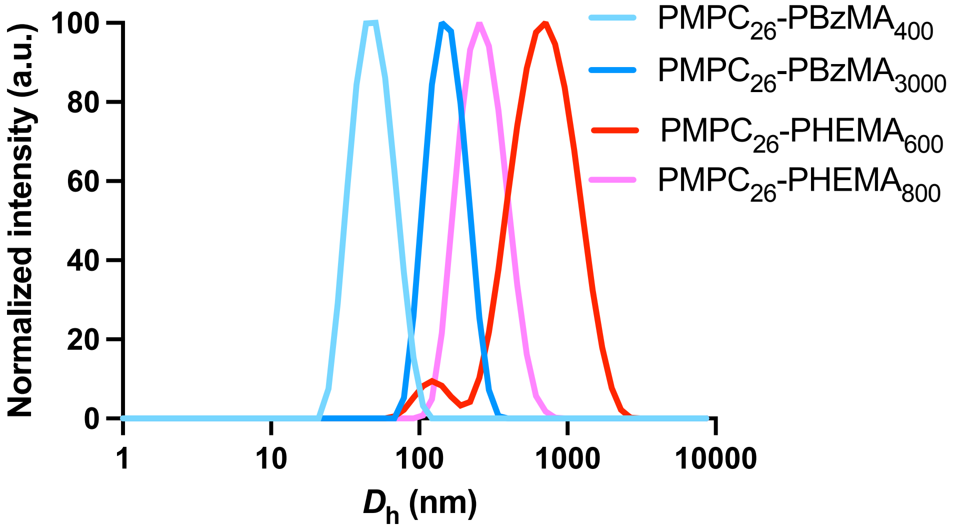


**Figure S6.** Intensity-weighted particle size distributions determined by DLS for PMPC$_{26}$-PHEMA$_n$ (n = 600, 800) and PMPC$_{26}$-PBzMA$_n$ (n = 400, 3000) diblock copolymer nanoparticles.

*Polymerisation of 2-acrylamido-2-methyl-1-propanesulfonic acid sodium salt using 4-cyano-4-(phenylcarbonothioylthio)pentanoic acid.* AMPS monomer (supplied as an 50% w/w aqueous solution, 15.2 g, 0.0736 mol, 50 eq), 4-cyano-4-(phenylcarbonothioylthio)pentanoic acid (PETTC) (150.0 mg, 0.44 mmol, 1 eq), and ACVA initiator (31.0 mg, 0.11 mmol, 0.25 eq, PETTC/ACVA molar ratio = 5.0) were dissolved in methanol (40 mL) to afford a 40% w/w solution in a sealed round-bottom flask containing a magnetic stir bar. This flask was deoxygenated with a stream of $N_2$ gas for 30 min. The flask was heated to 70 °C with magnetic stirring for 2 hours, and then the AMPS polymerisation was quenched by exposing the reaction

mixture to air while cooling the flask to 20 °C. A final AMPS conversion of 80% was determined by comparing the integrated vinyl proton signal at 5.65-6.20 ppm with the -C$H_2$-$SO_3$ signals assigned to the polymerised PAMPS units at 3.0–3.7 ppm using $^1$H NMR spectroscopy. The crude PAMPS homopolymer was dialysed for five days (MWCO = 3.5 kDa) and lyophilised overnight to afford a pale yellow solid. The mean degree of polymerisation (and hence $M_{n,\ NMR}$) was calculated by end-group analysis by comparing the integrated phenyl end group (t, 7.95 ppm) with the two -C$H_2$-$SO_3$ proton signals assigned to the polymerised PAMPS side chain at 3.0–3.7 ppm. $M_{n,\ NMR}$ = 9,300 Da ($DP_{PAMPS}$ = 39). SEC analysis (10 mM LiBr in DMF) indicated an $M_n$, of 7,500 Da and a $M_w/M_n$ of 1.35.

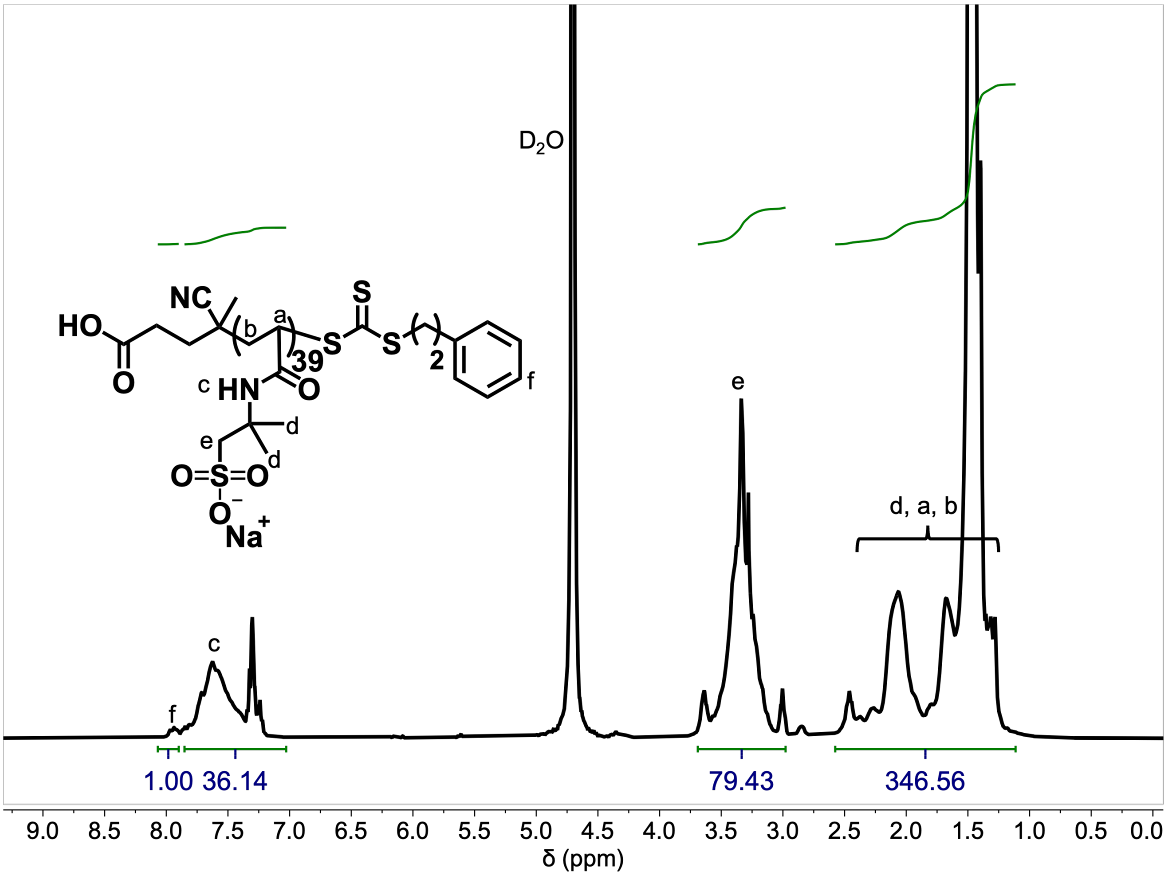


**Figure S7.** $^1$H NMR spectrum ($D_2O$, 400 MHz) recorded for the $PAMPS_{39}$ precursor.

*Synthesis of $PAMPS_{39}$-$PDAAM_n$ nanoparticles by RAFT aqueous dispersion polymerisation in presence of 0.1 M NaCl.* A typical synthetic protocol for the preparation of *$PAMPS_{39}$-$PDAAM_{250}$* nanoparticles at 10% w/w solids by RAFT aqueous dispersion polymerisation in 0.1 M NaCl is as follows. To a 6 mL glass vial containing a stirring bar, $PAMPS_{39}$ precursor (50 mg, 0.00539 × $10^{-3}$ mmol, 1 eq), DAAM (228 mg, 1.35 mmol, 250 eq) and VA-044 (0.4 mg, 0.001 × $10^{-3}$ mmol, 0.25 eq) were added and dissolved in 0.1 M NaCl (2.51 mL). The vial

was sealed with a rubber septum and the reaction solution was purged with $N_2$ gas for 20 min. The vial was then placed into an aluminum heating block that had been pre-heated to 50 °C. After 18 h, the polymerisation was quenched by immersing the glass vial in liquid nitrogen and exposing its contents to air. An aliquot was removed for $^1$H NMR spectroscopy studies in $CD_3OD$ and SEC analysis in DMF. TEM, DLS and ζ-potential analyses were performed on samples after dilution to an appropriate copolymer concentration. This synthetic protocol was repeated for [DAAM]/[$PAMPS_{39}$] molar ratio of 39 and 125. In each case, relatively high monomer conversions were confirmed by $^1$H NMR analysis, as indicated by the disappearance of the DAAM vinyl signals at 5.65-6.20 ppm.

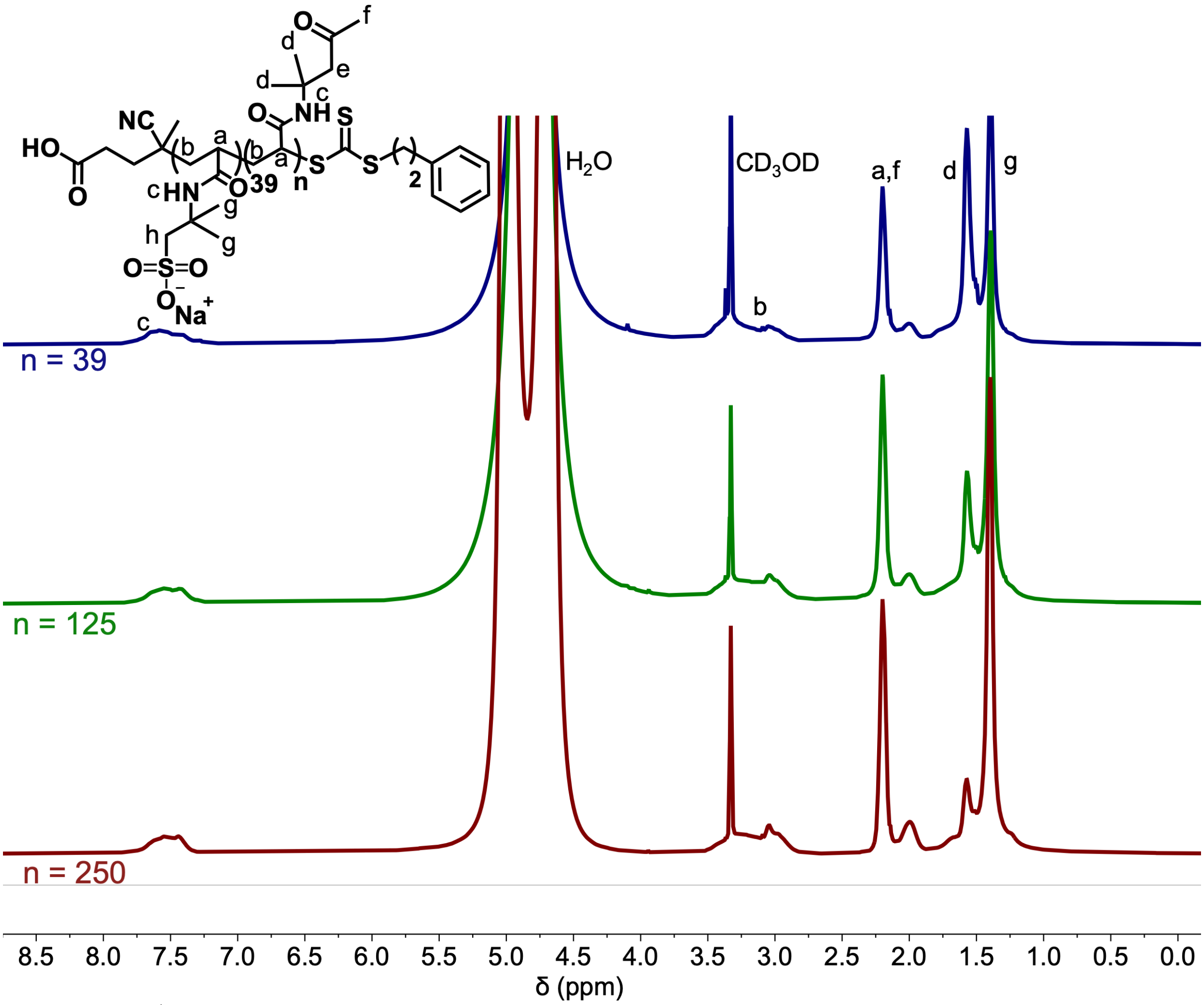


**Figure S8.** $^1$H NMR spectra ($CD_3OD$, 400 MHz) recorded for $PAMPS_{39}$-$PDAAM_n$ (n = 39, 125, 250) diblock copolymers prepared by RAFT aqueous dispersion polymerisation of DAAM when targeting 10% w/w solids using a $PAMPS_{39}$ precursor.

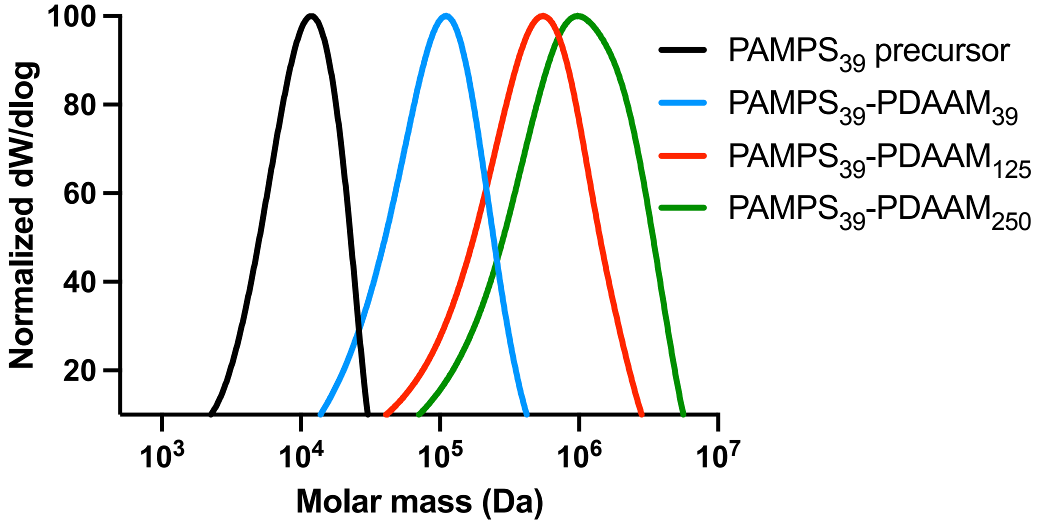


**Figure S9.** Normalised DMF SEC curves (refractive index detector) recorded for a series of $PAMPS_{39}$-$PDAAM_n$ (n = 39, 125, 250) diblock copolymers and the corresponding $PAMPS_{39}$ homopolymer precursor (black trace).

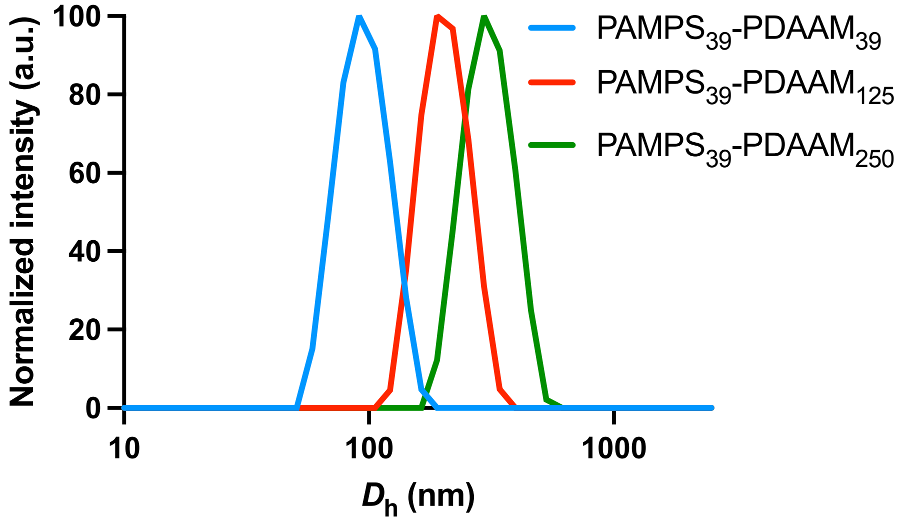


**Figure S10.** Intensity-weighted particle size distributions determined by DLS for a series of $PAMPS_{39}$-$PDAAM_n$ (n = 39, 125, 250) diblock copolymer nanoparticles.

*Polymerisation of [2-(methacryloyloxy)ethyl]trimethylammonium chloride using 4-cyano-4-(phenylcarbonothioylthio)pentanoic acid.* METAC monomer (supplied as an 80% w/w aqueous solution; 15.05 g, 0.073 mol, 150 eq), PETTC (0.164 g, 0.48 mmol; 1 eq), ACVA initiator (0.034 g, 0.12 mmol; PETTC/ACVA molar ratio = 5.0) were dissolved in methanol (38.0 mL) to afford a 40% w/w solution in a sealed round-bottom flask containing a magnetic stir bar. This flask was deoxygenated with a stream of $N_2$ gas for 30 min. The flask was heated to 70 °C with magnetic stirring for 2 hours, and then the METAC polymerisation was quenched by exposing the reaction mixture to air while cooling the flask to 20 °C. $^1$H NMR studies

indicated a METAC conversion of 82%, as judged by comparing the integrated vinyl proton signals at 5.7 and 6.1 ppm to the overlapping oxymethylene proton signals assigned to both PMETAC and METAC at 4.2-4.6 ppm. The crude PMETAC precursor was purified by precipitation into excess acetonitrile, redissolved in deionised water, and precipitated once more in acetonitrile. The purified PMETAC was redissolved in deionised water, dialysed for two days, and then lyophilised overnight to produce a pale-yellow powder. The mean degree of polymerisation (and hence $M_{n,\ NMR}$) was calculated by end-group by comparing the integrated phenyl end group (t, 7.95 ppm) to methyl protons (m, 0.75-1.25) of the polymer backbone. $M_{n,\ NMR}$ = 27,700 Da ($DP_{PMETAC}$ = 123). Aqueous SEC analysis indicated an $M_{n,}$ of 8,900 Da and a $M_w/M_n$ of 1.19.

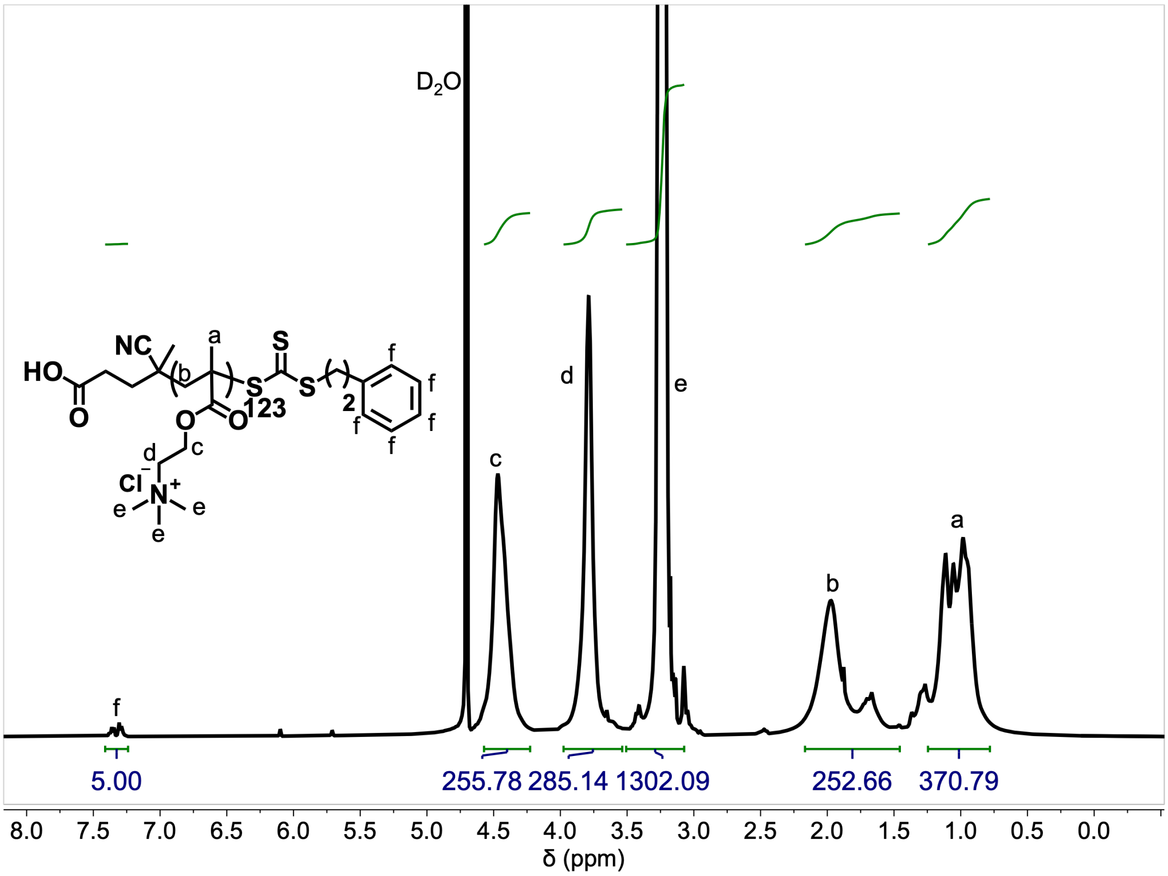


**Figure S11.** $^1H$ NMR spectrum ($D_2O$, 400 MHz) recorded for the $PMETAC_{123}$ precursor.

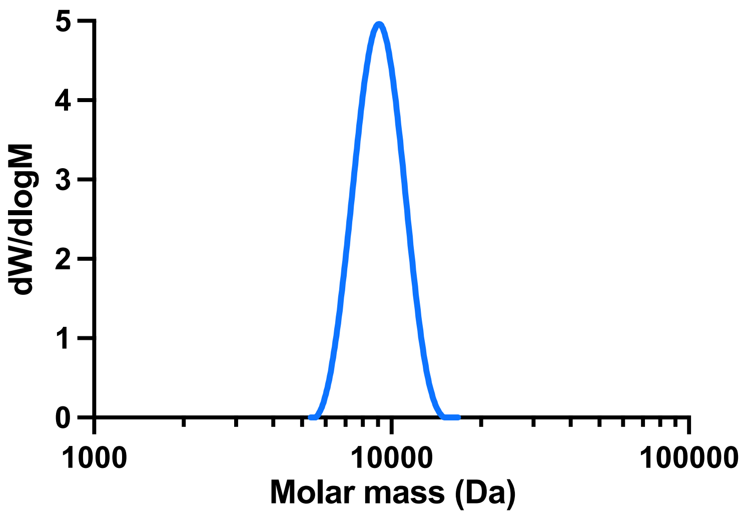


**Figure S12.** Aqueous SEC curve (refractive index detector) recorded for $PMETAC_{123}$ precursor.

*Synthesis of $PMETAC_{123}$-PBzMA and $PMETAC_{123}$-PBzA nanoparticles by RAFT aqueous emulsion polymerisation of BzMA or BzA in presence of 0.1 M NaCl.* A typical synthetic protocol for the preparation of *$PMETAC_{123}$-$PBzMA_{100}$* nanoparticles at 10% w/w solids by RAFT aqueous emulsion polymerisation in 0.1 M NaCl is as follows. To a 6 mL glass vial containing a stirring bar, $PMETAC_{123}$ precursor (50 mg, 0.0018 × $10^{-3}$ mmol, 1 eq), BzMA (146 mg, 0.9 mmol, 100 eq) and VA-050 (0.1 mg, 0.0005 × $10^{-3}$ mmol, 0.25 eq) were added and dissolved in 0.1 M NaCl (1.77 mL). The vial was sealed with a rubber septum, and the reaction solution was purged with $N_2$ gas for 20 min. The vial was then placed into an aluminum heating block that had been pre-heated to 60 °C. After 18 h, the polymerisation was quenched by immersing the glass vial in liquid nitrogen and exposing its contents to air. An aliquot was removed for $^1$H NMR spectroscopy studies in $(CD_3)_2SO$. TEM, DLS and ζ-potential analyses were performed on samples after dilution to an appropriate copolymer concentration. This synthetic protocol was repeated for [BzMA]/[$PMETAC_{123}$] molar ratio of 500, 1000, 2000 and [BzA]/[$PMETAC_{123}$] molar ratio of 100, 500, 1000, 2000. Both $PMETAC_{123}$-PBzMA and $PMETAC_{123}$-PBzA diblock copolymer series proved to be insoluble in common SEC solvents (e.g., DMF, THF, chloroform), which precluded determination of the final molar mass and copolymer dispersity determination. More than 99% BzMA and BzA conversion was achieved within 18 h at 70 °C, as judged by the complete disappearance of the methacrylic vinyl signals at 5.6-6.1 ppm.

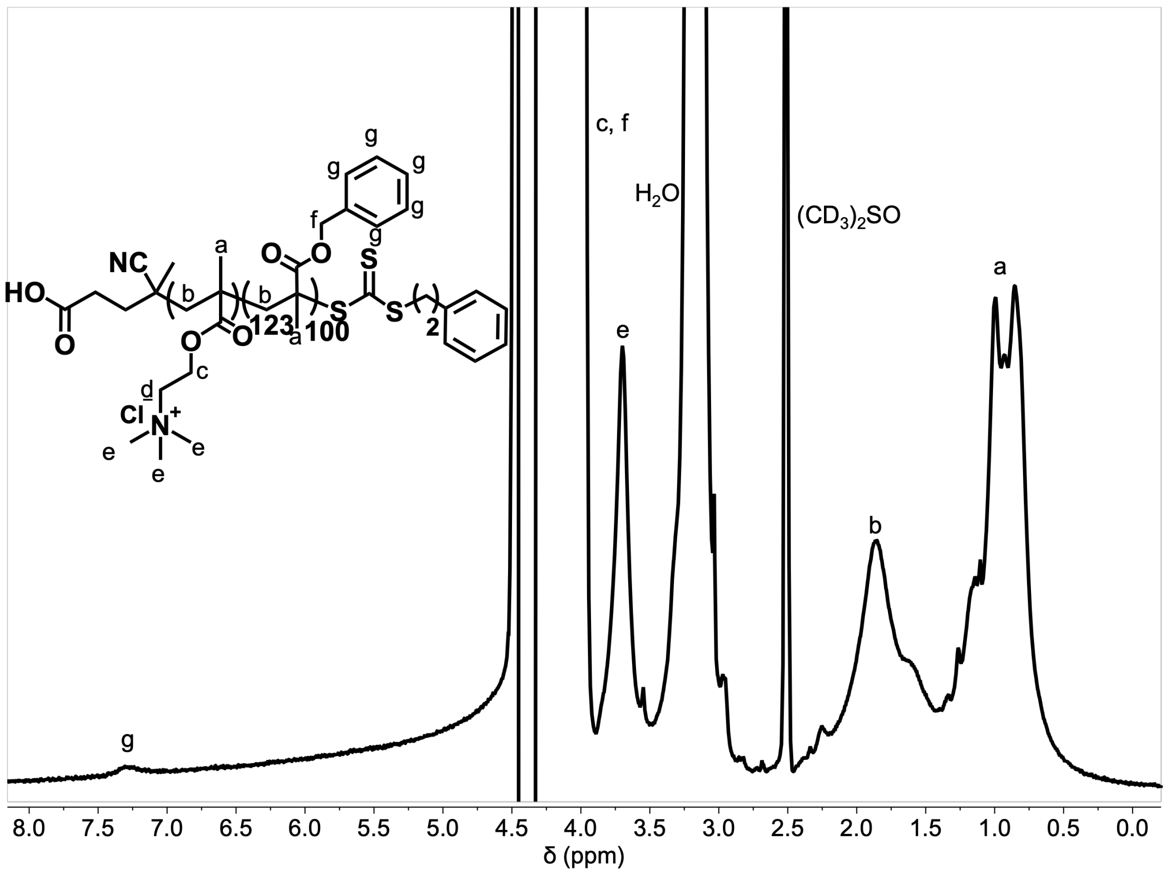


**Figure S13.** $^{1}$H NMR spectrum ((CD$_3$)$_2$SO, 400 MHz) recorded for the PMETAC$_{123}$-PBzMA$_{100}$.

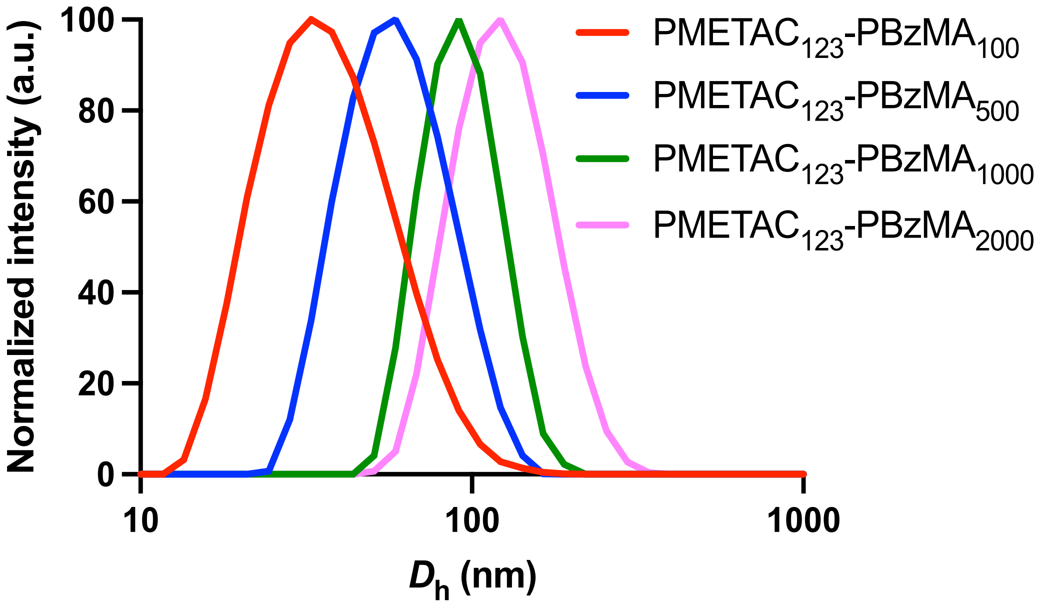


**Figure S14.** Intensity-weighted particle size distributions determined by DLS for a series of PMETAC$_{123}$-PBzMA$_n$ (n = 100, 500, 1000, 2000) diblock copolymer nanoparticles.

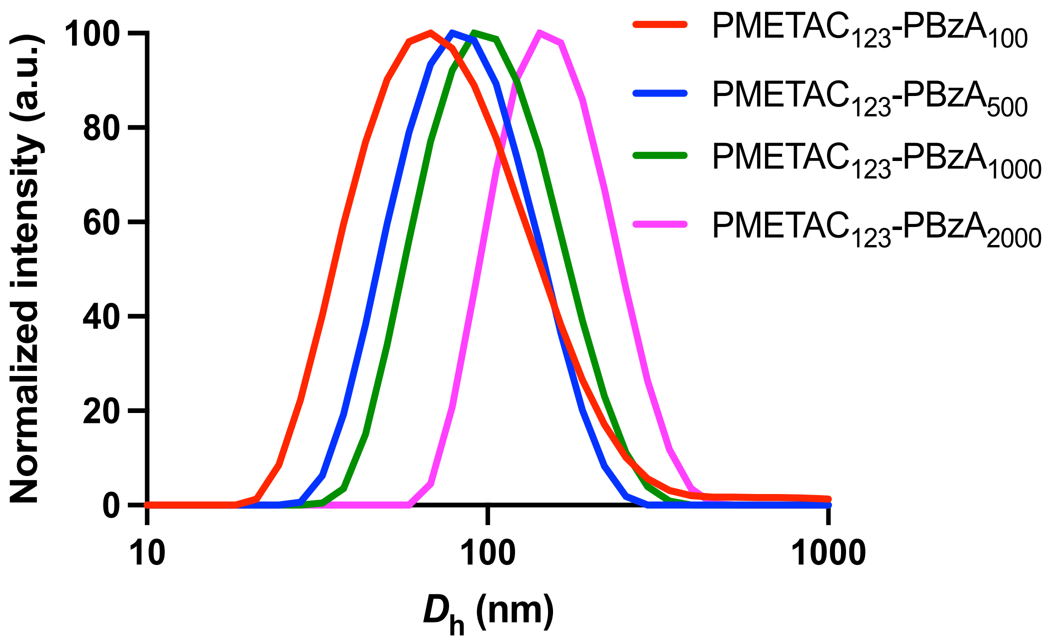


**Figure S15.** Intensity-weighted particle size distributions determined by DLS for a series of $PMETAC_{123}$-$PBzA_n$ (n = 100, 500, 1000, 2000) diblock copolymer nanoparticles.

*Synthesis of $PMETAC_{123}$-PBzA-PEGDMA core-crosslinked nanoparticles by RAFT aqueous emulsion polymerisation of EGDMA in presence of 0.1 M NaCl.* A typical synthetic protocol for the preparation of *$PMETAC_{123}$-$PBzA_{100}$-$PEGDMA_5$ core-crosslinked* nanoparticles at 10% w/w solids by RAFT aqueous emulsion polymerisation in 0.1 M NaCl is as follows. To a 6 mL glass vial containing a stirring bar, 1 mL of 10% w/w *$PMETAC_{123}$-$PBzA_{100}$* nanoparticles (100 mg, 0.0023 × $10^{-3}$ mmol, 1 eq), EGDMA (2.2 mg, 0.012 mmol, 5 eq) and VA-050 (0.1 mg, 0.0006 × $10^{-3}$ mmol, 0.25 eq) were added and diluter with 240 µL of 0.1 M NaCl (total volume 1.24 mL). The vial was sealed with a rubber septum, and the reaction solution was purged with $N_2$ gas for 20 min. The vial was then placed into an aluminum heating block that had been pre-heated to 60 °C. After 18 h, the polymerisation was quenched by immersing the glass vial in liquid nitrogen and exposing its contents to air. Due to cross-link nature of triblock copolymer nanoparticles, $^1$H NMR (assumed >99% after 18 h) and SEC analysis was not possible. TEM and DLS analyses were performed on samples after dilution to an appropriate copolymer concentration. All block copolymer dispersions were dialysed for 48 h to remove salt and lyophilised overnight prior to DSC analysis. This synthetic protocol was repeated for targeted compositions of $PMETAC_{123}$-$PBzA_{500}$-$PEGDMA_{25}$, $PMETAC_{123}$-$PBzA_{1000}$-$PEGDMA_{50}$ and $PMETAC_{123}$-$PBzA_{2000}$-$PEGDMA_{100}$.

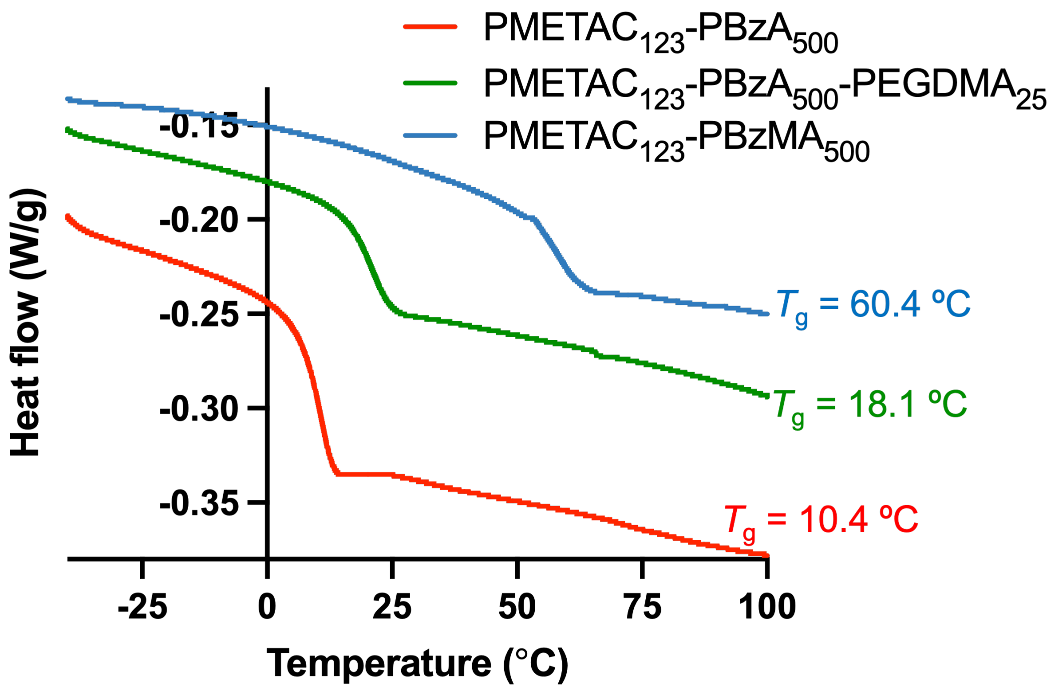


**Figure S16.** DSC curves recorded for $PMETAC_{123}$-$PBzA_{500}$ (red trace), $PMETAC_{123}$-$PBzA_{500}$-$PEGDMA_{25}$ (green trace) and $PMETAC_{123}$-$PBzMA_{500}$ (blue trace) at a heating rate of 10 °C per min.

## Supporting Ice Recrystallisation Inhibition/ Ice Shaping Data

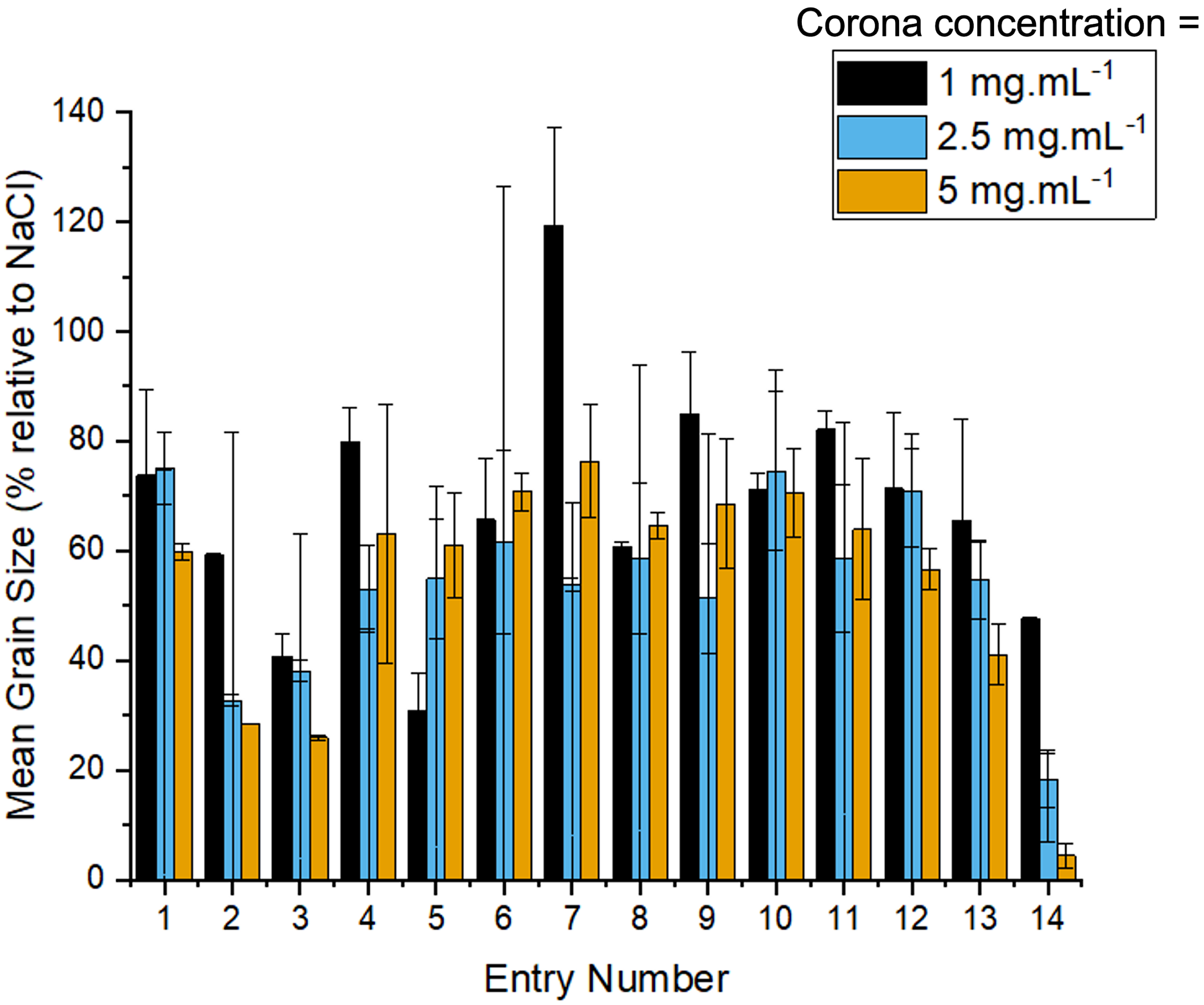


**Figure S17.** Ice recrystallisation inhibition (IRI) activity summary of neutrally charged (entries 1-5) block copolymer nanoparticles, negatively charged (entries 6-9) block copolymer nanoparticles and positively charged block copolymer nanoparticles with high $T_g$ cores based on benzyl methacrylate (entries 11-14) at corona concentrations of 5, 2.5 and 1 mg/mL.

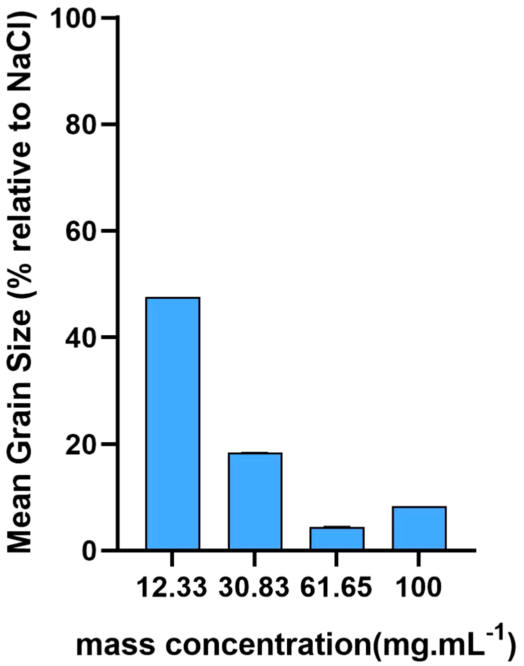


**Figure S18.** IRI activity summary of PMETAC$_{123}$-PBzMA$_{2000}$ nanoparticles corrected to total mass [= solids] concentration. Error bars are ± SD from a minimum of three repeats. The percent mean grain size (MGS) was reported relative to saline control ([NaCl] = 0.1 M).

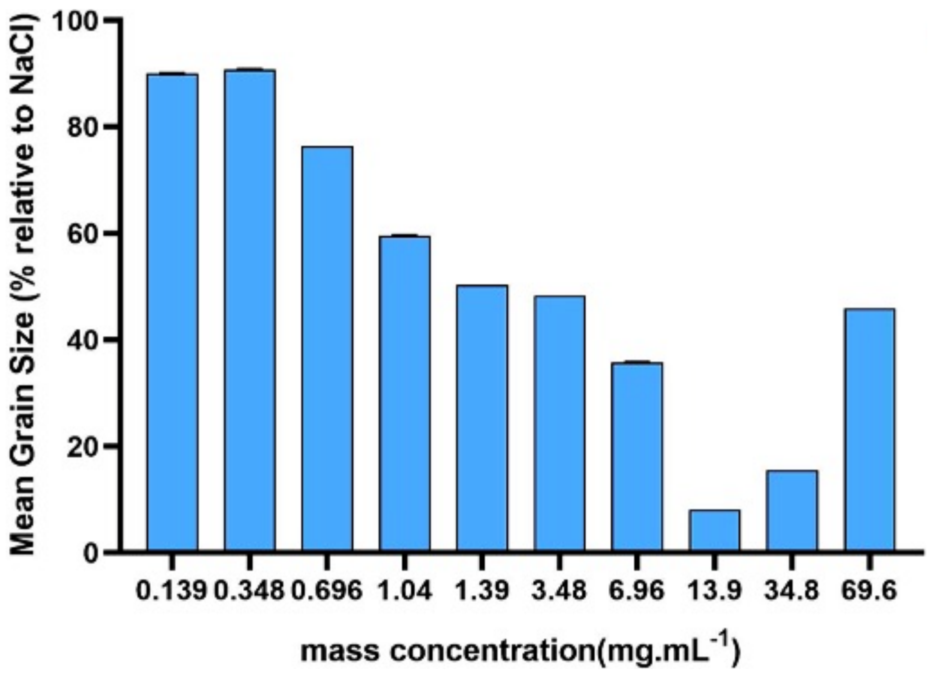


**Figure S19.** IRI activity summary of PMETAC$_{123}$-PBzA$_{2000}$ nanoparticles corrected to total mass [= solids] concentration. Error bars are ± SD from a minimum of three repeats. The percent mean grain size (MGS) was reported relative to saline control ([NaCl] = 0.1 M).

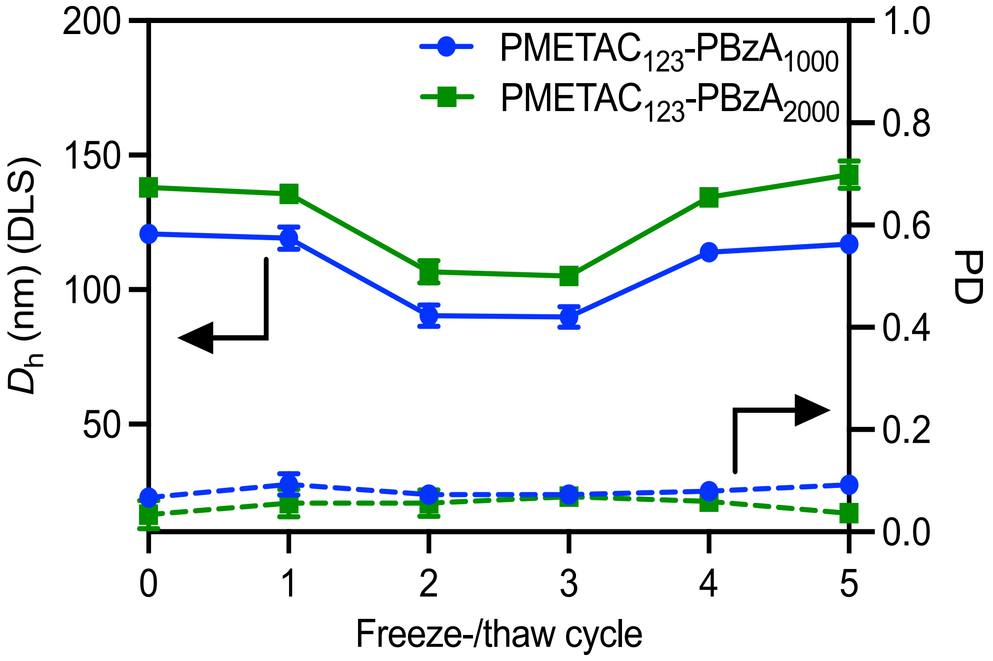


**Figure S20.** Colloidal stability studies by monitoring hydrodynamic diameter ($D_h$) and polydispersity (PD) by DLS for PMETAC$_{123}$-PBzA$_{1000}$ (blue trace) and PMETAC$_{123}$-PBzA$_{2000}$ (green trace) nanoparticle solutions (1% w/w) upon five freeze-thaw cycles (the error bars show the standard deviation from 5 repeat measurements).

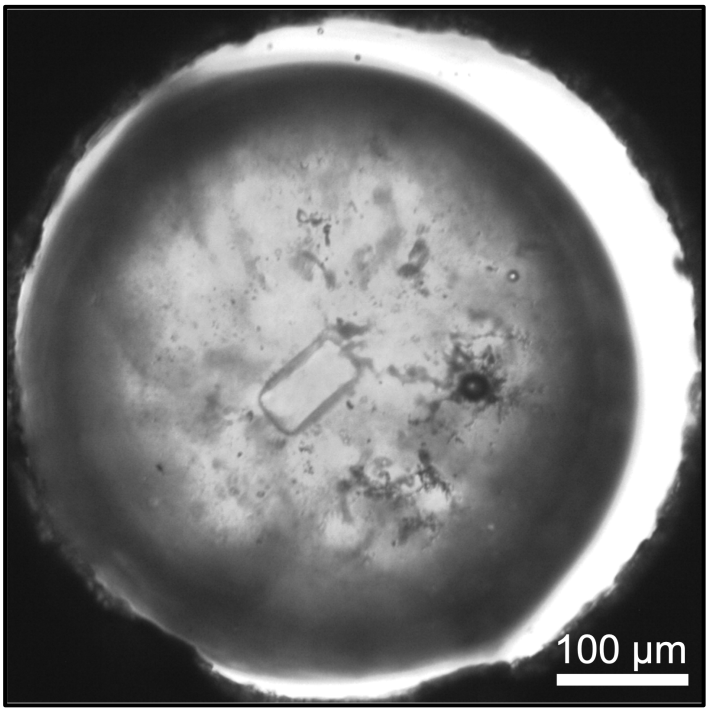


**Figure S21.** Micrographs of ice crystal growth using nanolitre osmometer for [PMETAC] = 0.3 mg/mL of $PMETAC_{123}$-$PBzMA_{2000}$ nanoparticles.

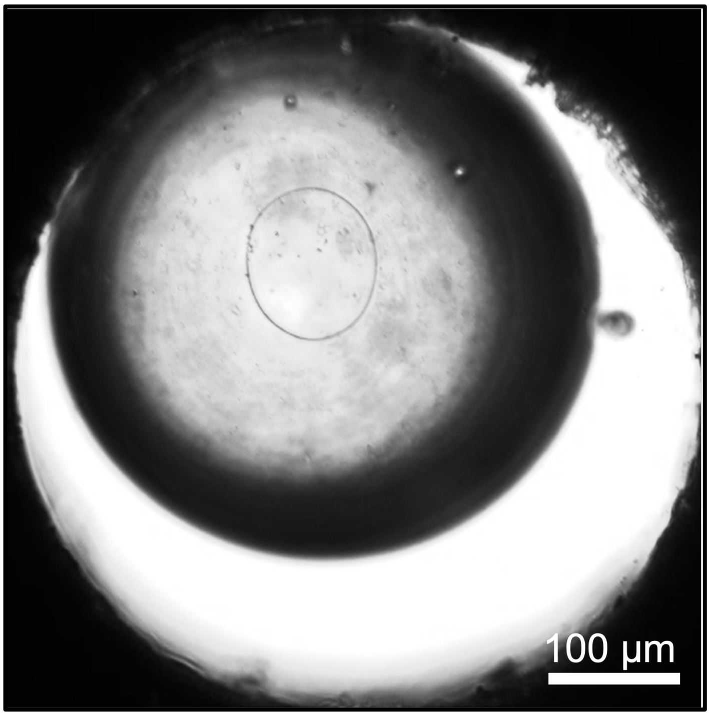


**Figure S22.** Micrographs of ice crystal growth using nanolitre osmometer for [PMETAC] = 0.3 mg/mL of $PMETAC_{123}$-$PBzA_{2000}$ nanoparticles.

## References


[1] E. R. Jones, M. Semsarilar, A. Blanazs, S. P. Armes, *Macromolecules* **2012**, *45*, 5091–5098.

[2] M. M. Tomczak, C. B. Marshall, J. A. Gilbert, P. L. Davies, *Biochem. Biophys. Res. Commun.* **2003**, *311*, 1041–1046.

[3] A. Kirillov, E. Mintun, N. Ravi, H. Mao, C. Rolland, L. Gustafson, T. Xiao, S. Whitehead, A. C. Berg, W. Y. Lo, P. Dollár, R. Girshick, *Proc. IEEE Int. Conf. Comput. Vis.* **2023**, 3992–4003.

[4] I. AU - Braslavsky, R. AU - Drori, *JoVE* **2013**, e4189.

[5] G. Bai, D. Gao, J. Wang, *Carbon N. Y.* **2017**, *124*, 415–421.

[6] J. C. Kasper, W. Friess, *Eur. J. Pharm. Biopharm.* **2011**, *78*, 248–263.

[7] D. L. Beattie, O. O. Mykhaylyk, A. J. Ryan, S. P. Armes, *Soft Matter* **2021**, *17*, 5602–5612.
